# Short branch attraction in phylogenomic inference under the multispecies coalescent

**DOI:** 10.3389/fevo.2023.1134764

**Published:** 2023-06-28

**Authors:** Liang Liu, Lili Yu, Shaoyuan Wu, Jonathan Arnold, Christopher Whalen, Charles Davis, Scott Edwards

**Affiliations:** 1 Department of Statistics and Institute of Bioinformatics, University of Georgia, Athens, GA, United States; 2 Department of Biostatistics, Georgia Southern University, Statesboro, GA, United States; 3 Jiangsu Key Laboratory of Phylogenomics and Comparative Genomics, Jiangsu International Joint Center of Genomics, School of Life Sciences, Jiangsu Normal University, Xuzhou, Jiangsu, China; 4 Department of Genetics, University of Georgia, Athens, GA, United States; 5 Department of Epidemiology and Biostatistics, College of Public Health, University of Georgia, Athens, GA, United States; 6 Department of Organismic and Evolutionary Biology, Harvard University, Cambridge, MA, United States

**Keywords:** coalescent methods, species trees, gene trees, multispecies coalescent model, long branch attraction, short branch attraction

## Abstract

Accurate reconstruction of species trees often relies on the quality of input gene trees estimated from molecular sequences. Previous studies suggested that if the sequence length is fixed, the maximum likelihood may produce biased gene trees which subsequently mislead inference of species trees. Two key questions need to be answered in this context: what are the scenarios that may result in consistently biased gene trees? and for those scenarios, are there any remedies that may remove or at least reduce the misleading effects of consistently biased gene trees? In this article, we establish a theoretical framework to address these questions. Considering a scenario where the true gene tree is a 4-taxon star tree $T^{\text{*}}=\left(S_{1},S_{2},S_{3},S_{4}\right)$ with two short branches leading to the species ${\text{S}}_{1}$ and ${\text{S}}_{2}$, we demonstrate that maximum likelihood significantly favors the wrong bifurcating tree $\left[\left({\text{S}}_{1},{\text{S}}_{2}\right),{\text{S}}_{3},{\text{S}}_{4}\right]$ grouping the two species ${\text{S}}_{1}$ and ${\text{S}}_{2}$ with short branches. We name this inconsistent behavior short branch attraction, which may occur in real-world data involving a 4-taxon bifurcating gene tree with a short internal branch. If no mutation occurs along the internal branch, which is likely if the internal branch is short, the 4-taxon bifurcating tree is equivalent to the 4-taxon star tree and thus will suffer the same misleading effect of short branch attraction. Theoretical and simulation results further demonstrate that short branch attraction may occur in gene trees and species trees of arbitrary size. Moreover, short branch attraction is primarily caused by a lack of phylogenetic information in sequence data, suggesting that converting short internal branches to polytomies in the estimated gene trees can significantly reduce artifacts induced by short branch attraction.

## 1. Introduction

Coalescent-based approaches have been shown to be statistically consistent in estimating species trees as the number of loci and the sequence length increase to infinity (Felsenstein, 2006; Liu et al., 2010). However, short sequences are commonly observed in the coding and non-coding regions across species in the Tree of Life, necessitating an assessment of coalescent approaches in the context of finite sequence lengths. Previous studies (Roch and Warnow, 2015; Roch et al., 2019) showed that molecular sequences with a fixed length can mislead coalescent methods to estimate an incorrect species tree even if the number of loci increases to infinity. The failure of coalescent methods in this case is not caused by the deficiency of coalescent models (i.e., violation of the coalescent model assumptions) (Carvajal-Rodriguez et al., 2006; Adams et al., 2018; Jiang et al., 2020), but by the maximum likelihood (ML) gene trees consistently favor incorrect phylogenetic relationships of the species involved. Specifically, molecular sequences with a finite length may produce biased gene trees. These biased gene trees can subsequently mislead coalescent methods to estimate an incorrect species tree. It should be noted here that random bias in gene tree estimation does not have a major effect on species tree estimation. For instance, if some loci support incorrect relationships between two species ${\text{S}}_{1}$ and ${\text{S}}_{2}$, while others support incorrect relationships between other species $S_{i}$ and $S_{\text{j}}$, the misleading effects of biased gene trees are canceled out in estimating species trees. However, the greatest challenge in species tree inference arises if gene tree inference is consistently biased towards supporting the same or a similar set of incorrect relationships. Key questions that need to be addressed are what scenarios result in consistently biased gene trees and if any remedies can alleviate the misleading effects. These questions do not have straightforward solutions. Finding both necessary and sufficient conditions for biased gene trees is challenging, and while large gene tree estimation errors may mislead species tree estimation, coalescent methods are robust to a certain degree of gene tree errors and can still recover the true species tree (Liu et al., 2015). This article aims to establish a theoretical framework to address these questions, enabling the identification of problematic scenarios in real-world data analysis and providing potential solutions to these issues.

Genes with minimal phylogenetic information can substantially increase gene tree estimation error, which may reduce the accuracy of species tree inference (Xi et al., 2015). Similar consequences involving minimal phylogenetic information have been observed for another well-documented phenomena long branch attraction (LBA) (Felsenstein, 1978), which can mislead phylogenetic tree inference as well. LBA occurs when two long terminal branches are separated by a short internal branch in a phylogenetic tree, another example of lack of phylogenetic signals causing systematic errors in estimating phylogenetic trees. Long branch artefacts have historically been seen as a major problem for parsimony-based inference, but maximum likelihood and Bayesian approaches can also be susceptible to these issues (Martyn and Steel, 2012; Su and Townsend, 2015; Susko, 2015). Current theories on LBA have identified branch length conditions that lead to inconsistent inferences when using maximum parsimony (Hendy and Penny, 1989; Kim, 1996). Moreover, Townsend et al. (2012) proposed a “signal and noise” framework that uses substitution rates to estimate the ability of molecular sequences to resolve a four-taxon tree with equally-subtending branch lengths. This framework can be generalized to account for LBA bias resulting from asymmetric topologies or unequal evolution rates and can identify branch length conditions where phylogenetic inference is inconsistent for these types of phylogenies (Su and Townsend, 2015). Nevertheless, these theories originated within the framework of conventional phylogenetic models (Felsenstein, 1981). However, when it comes to the multispecies coalescent model (Rannala and Yang, 2003), there is a scarcity of theories addressing the specific conditions of branch lengths that can mislead species tree estimation. A review of the evidence on the impact of missing data on species tree estimation indicates that missing data can bias both gene tree and species tree estimation (Xi et al., 2016). The estimation problems induced by minimal phylogenetic signal can be alleviated by sampling more informative gene sequences. More severe challenges arise, however, when gene tree estimation is biased and cannot be remedied by increasing the sequence length or the number of loci (Xi et al., 2015).

Owing to its asymptotic properties (i.e., consistency, asymptotic unbiasedness, asymptotic efficiency, etc.), ML is among the most popular methods for building phylogenetic trees from molecular sequence data. It is well known that maximum likelihood estimates (MLE) are often biased if the sample size is finite (Mardia et al., 1999). Given a set $\left(x_{1},\ldots ,x_{n}\right)$ of identically and independently distributed random variables generated from the normal distribution with mean ∝ and variance $\sigma^{2}$, the MLE of the variance $\sigma^{2}$ is ${\hat{\sigma }}_{MLE}^{2}=\frac{1}{n}\sum_{i=1}^{n}{\left(x_{i}-\bar{x}\right)}^{2}$. As $E\left({\hat{\sigma }}_{MLE}^{2}\right)=\frac{n-1}{n}\sigma^{2}$, the MLE ${\hat{\sigma }}_{MLE}^{2}$ underestimates the variance $\sigma^{2}$. Biasness is a good measure of an estimator’s performance in a finite sample size. A continuous estimator $\hat{\theta }$ of the parameter θ is said to be unbiased if its expectation is equal to the parameter value, i.e., $E\left(\hat{\theta }\right)=\theta$. Since the tree topology is a discrete random variable, the ML tree $\hat{T}$ is defined as an unbiased estimator of the true tree $T^{\text{*}}$ if the most probably ML tree is the true tree $T^{\text{*}}$, i.e., $P\left(\hat{T}=T^{\text{*}}\right)>P\left(\hat{T}=T\right)$ for any $T\neq T^{\text{*}}$. In contrast to previous studies (Su and Townsend, 2015) that employed predicted utility to identify branch length conditions in which ML methods fail to recover the true phylogenetic tree, this study takes a different approach to investigate the statistical properties, specifically the bias, of ML phylogenetic trees. In this paper, we can show that under certain conditions ML trees are biased estimators of the underlying phylogenetic trees (i.e., gene trees and species trees) if the sequence length is finite. The proof begins with a scenario where the true gene tree is a 4-taxon star tree $T^{\text{*}}=\left(S_{1},S_{2},S_{3},S_{4}\right)$ with two short branches leading to species $S_{1}$ and $S_{2}$. In this case, ML significantly favors the wrong bifurcating tree $\left(\left(S_{1},S_{2}\right),S_{3},S_{4}\right)$ grouping the two species $S_{1}$ and $S_{2}$ with short branches, which is called short branch attraction (SBA). SBA may also occur in a 4-taxon bifurcating gene tree with a short internal branch. If no mutation occurs in the internal branch, which is quite likely if the internal branch is short, the 4-taxon bifurcating tree is equivalent to the 4-taxon star tree and thus it suffers the same misleading effect of short branch attraction. Similarly, if the true species tree is a 4-taxon star tree with two short branches leading to the species $S_{1}$ and $S_{2}$, most gene trees generated from this species tree are the 4-taxon bifurcating trees with a short internal branch. Due to SBA, the ML gene trees consistently favor the wrong tree $\left[\left(S_{1},S_{2}\right),S_{3},S_{4}\right]$ across genes, which subsequently mislead the coalescent methods (Liu and Yu, 2011; Mirarab and Warnow, 2015) to estimate the wrong species tree $\left(\left(S_{1},S_{2}\right),S_{3},S_{4}\right)$. Our findings indicate that a phylogenetic tree may exhibit the short branch artifact when a quartet within the tree consists of a short internal branch and four unequal external branches. As such, the phenomenon known as long branch attraction, which is characterized by a short internal branch and dissimilar external branches, represents a specific instance of the aforementioned artifact. This study presents a comprehensive analytical framework for examining the impact of branch length heterogeneity on the inference of gene and species trees using finite sequence lengths.

## 2. Results

### 2.1. Biased maximum likelihood estimates of phylogenetic trees

The MLEs of phylogenetic trees have been shown to be statistically consistent as the sequence length K goes to infinity (Rogers, 1997), i.e., $P\left({\hat{T}}_{MLE}=T^{\text{*}}\right)\to 1$ as K→∞, where ${\hat{T}}_{MLE}$ is the MLE of the phylogenetic tree $T^{\text{*}}$. If the sequence length K is finite, ML methods may produce a biased estimate of the phylogenetic tree $T^{\text{*}}$. Consider a 4-taxon tree with an internal branch of length $t_{0}\geq 0$ and four terminal branches of lengths $t_{1}$, $t_{2}$, $t_{3}$, $t_{4}$ in mutation units (i.e., the branch length t represents the number of mutations per site). Without loss of generality, we assume that $0\leq t_{1}\leq t_{2}\leq t_{3}\leq t_{4}<\infty$. Let $D=\left\{d_{1},\ldots ,d_{K}\right\}$ be the DNA alignment of length K generated from the 4-taxon tree. In this paper, the Jukes-Cantor substitution model (Jukes and Cantor, 1969) is adopted for modeling the evolution of a single nucleotide. Under the Jukes-Cantor model, there are 15 site patterns with distinct probabilities – a pattern xxxx for 4 identical nucleotides, seven patterns xxxy, xxyx, xyxx, yxxx, xxyy, xyxy, xyyx for two different nucleotides, six patterns xxyz, xyxz, xyzx, yxxz, yxzx, yzxx for three different nucleotides, and a single pattern xyzw for four different nucleotides. Let $\omega =\left\{\omega_{1},\ldots ,\omega_{15}\right\}$ be the frequencies of 15 site patterns in the sequence alignment D. The frequencies ω follow the multinomial distribution with the probabilities $p=\left\{p_{1},\ldots ,p_{15}\right\}$ of 15 site patterns, subject to a constraint $\sum_{i}\omega_{i}=K$, i.e.,

(1) $$P\left(\omega |p\right)=\frac{K!}{\omega_{1}!\ldots \omega_{15}!}p_{1}^{\omega_{1}}\ldots p_{15}^{\omega_{15}}$$

The probabilities $p=\left\{p_{1},\ldots ,p_{15}\right\}$ of 15 site patterns are functions of the true 4-taxon tree $T^{\text{*}}$ and branch lengths $t=\left\{t_{0},t_{1},t_{2},t_{3},t_{4}\right\}$. The probability of the alignment D is equal to the probability of frequencies ω, i.e., $P\left(D|T^{\text{*}},t\right)=P\left(\omega |p\right)$. If the sequence length is K, the number of different alignments is $15^{k}$. For simplicity, we assume that the MLE is unique. Then the ML tree can be obtained from each of the $15^{k}$ alignments. Let $T_{1}=\left(\left(S_{1},S_{2}\right),S_{3},S_{4}\right)$, $T_{2}=\left(\left(S_{1},S_{3}\right),S_{2},S_{4}\right)$, and $T_{3}=\left(\left(S_{1},S_{4}\right),S_{2},S_{3}\right)$ be the three (unrooted) ML trees estimated from the sequence alignment D. The probability that the MLE $T_{MLE}$ is equal to the tree $T=\left\{T_{j},j=1,2,3\right\}$ is given by

(2) $$P\left({\hat{T}}_{MLE}=T_{j}\right)=\sum_{i}P\left(D_{i}|,T^{\text{*}}|,t\right)$$

Here $D_{i}$ ’s are the alignments from which the MLE tree is $T_{j}$. The MLE ${\hat{T}}_{MLE}$ is said to be biased if the most probable ML tree is not the true tree $T^{\text{*}}$, i.e., $P\left({\hat{T}}_{MLE}=T_{j}\right)>P\left({\hat{T}}_{MLE}=T^{\text{*}}\right)$ for some $T_{j}\neq T^{\text{*}}$.

#### 2.1.1. Short branch attraction In gene trees

In this section, we analyze three distinct scenarios (4-taxon star trees, 4-taxon bifurcating trees, and n-taxon trees) for gene trees. We show that the SBA artifact can mislead the ML estimation of gene trees in these scenarios.

##### 2.1.1.1. Scenario 1: 4-taxon star trees

**FIGURE 1:**
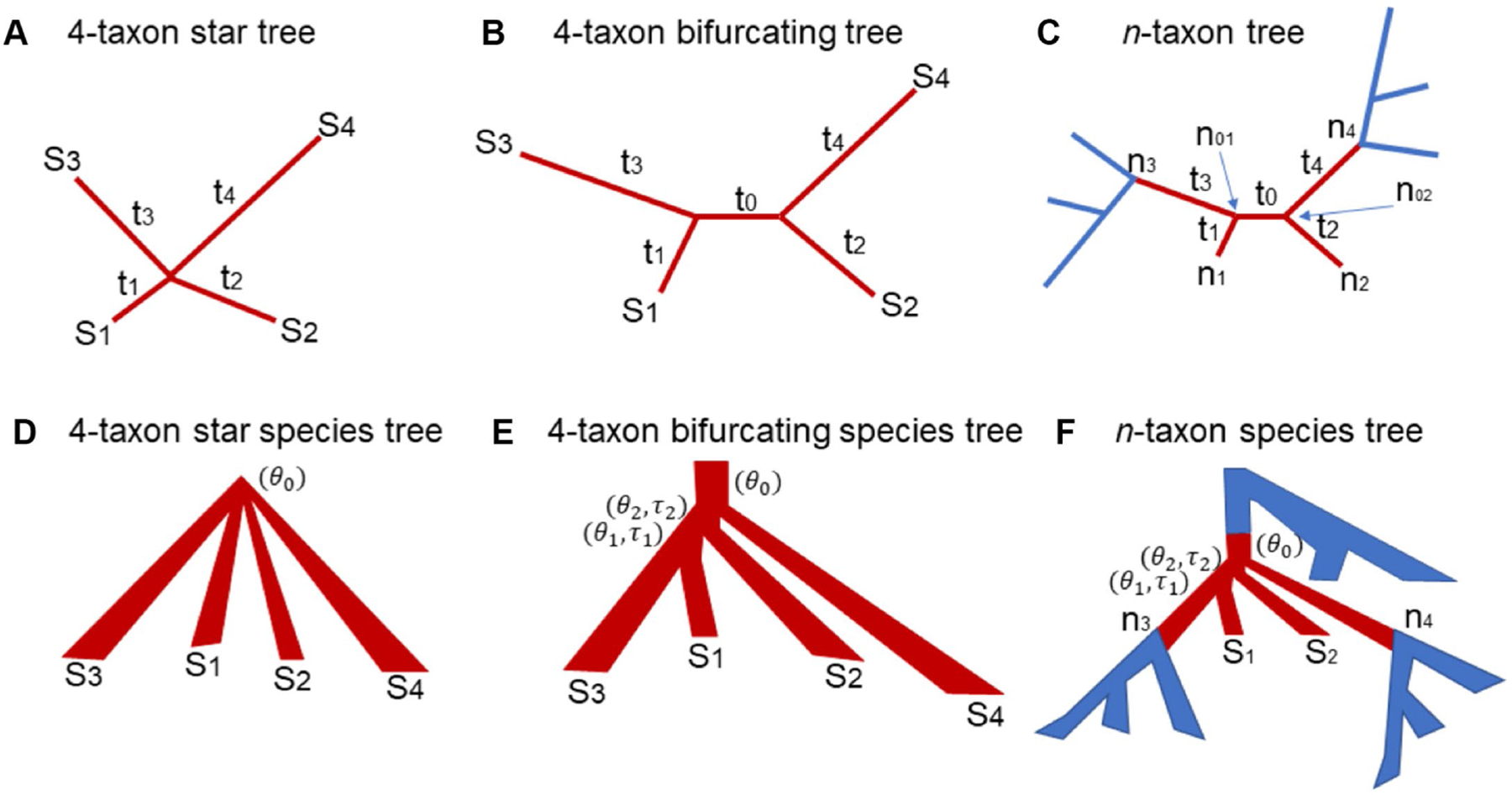
The 4-taxon gene trees and species trees used in the theoretical proof and simulation. **(A)** 4-taxon star tree: $t_{1}$, $t_{2}$, $t_{3}$, $t_{4}$ are the lengths of the four terminal branches leading to the species ${\text{S}}_{1}$, ${\text{S}}_{2}$, ${\text{S}}_{3}$, and ${\text{S}}_{4}$. **(B)** 4-taxon bifurcating tree: $t_{0}$ is the length of the internal branch and $t_{1}$, $t_{2}$, $t_{3}$, $t_{4}$ are the lengths of the four terminal branches leading to the species ${\text{S}}_{1}$, ${\text{S}}_{2}$, ${\text{S}}_{3}$, and ${\text{S}}_{4}$. **(C)** n-taxon bifurcating tree: the 4-taxon subtree is highlighted in red. The subtree has two internal nodes $n_{01}$ and $n_{02}$ and $t_{0}$, $t_{1}$, $t_{2}$, $t_{3}$, $t_{4}$ are the branch lengths of the subtree. **(D)** 4-taxon star species tree: $\theta_{0}$ is the population size parameter in the root population. **(E)** 4-taxon bifurcating species tree: $\theta_{0}$ is the population size parameter in the root population. $\theta_{1}$ and $\tau_{1}$ are the population size parameter and the branch length of the ancestral population of the species ${\text{S}}_{1}$ and ${\text{S}}_{2}$, while $\theta_{2}$ and $\tau_{2}$ are the population size parameter and the branch length of the ancestral population of the species ${\text{S}}_{1}$, ${\text{S}}_{2}$ and ${\text{S}}_{3}$. **(F)** n-taxon species tree: the 4-taxon subtree is highlighted in red.

We first consider a 4-taxon star tree where the four species $S_{1}$, $S_{2}$, $S_{3}$, $S_{4}$ diverged from the same ancestral node (Figure 1A). Let W, X, Y, Z be the nucleotides of the species $S_{1}$, $S_{2}$, $S_{3}$, $S_{4}$ in a single site of the DNA alignment D. Let H be the nucleotide at the internal node of the star tree $T^{\text{*}}$. Given the nucleotide H, the probability of nucleotides W, X, Y, Z is the multiplication of the probabilities of four terminal branches, i.e.,

(3) $$P\left(WXYZ|H,T^{\text{*}},t_{1},t_{2},t_{3},t_{4}\right)=P_{HW}\left(t_{1}\right)P_{HX}\left(t_{2}\right)P_{HY}\left(t_{3}\right)P_{HZ}\left(t_{4}\right)$$

In (3), $P_{HW}\left(t_{1}\right)$ is the probability that the nucleotide H changes to the nucleotide W after time $t_{1}$. Because $P\left(H\right)=\frac{1}{4}$ for H=A,C,G,T, the probability of a single site for a star tree $T^{\text{*}}$ is given by

(4) $$\begin{array}{l} P\left(WXYZ|T^{\text{*}},t_{1},t_{2},t_{3},t_{4}\right)\\ =\frac{1}{4}\sum_{H=A,C,G,T}P_{HW}\left(t_{1}\right)P_{HX}\left(t_{2}\right)P_{HY}\left(t_{3}\right)P_{HZ}\left(t_{4}\right) \end{array}$$

Additionally, there are 12 out of 15 nucleotide patterns that produce an unresolved ML tree (a star tree, i.e., ${\hat{T}}_{MLE}=T^{\text{*}}$). The remaining three patterns (xxyy, xyxy, xyyx) lead to a bifurcating ML tree. If the sequence length K=1, it follows from equation (2) that the probability $P\left({\hat{T}}_{MLE}=T^{\text{*}}\right)$ that the MLE is a star tree is equal to the sum of the probabilities of the 12 site patterns, and the probability that the MLE is a bifurcating tree, which is the true tree $T^{\text{*}}$, is given by $P\left({\hat{T}}_{MLE}\neq T^{\text{*}}\right)=P\left(xxyy\right)+P\left(xyxy\right)+P\left(xyyx\right)$. Because $P\left(xxxy\right)+P\left(xxyx\right)+P\left(xyxx\right)+P\left(yxxx\right)>P\left(xxyy\right)+P\left(xyxy\right)+P\left(xyyx\right)$ (Supplementary Appendix A1), the probability $P\left({\hat{T}}_{MLE}=T^{\text{*}}\right)$ that the MLE ${\hat{T}}_{MLE}$ is the true star tree $T^{\text{*}}$ is greater than the probability $P\left({\hat{T}}_{MLE}\neq T^{\text{*}}\right)$ that the MLE ${\hat{T}}_{MLE}$ is not the true star tree $T^{\text{*}}$. Thus, the ML tree ${\hat{T}}_{MLE}$ is an unbiased estimator of the 4-taxon star tree $T^{\text{*}}$. In real data analysis, most phylogenetic programs, for example RAxML (Stamatakis et al., 2005) and PHYML (Guindon et al., 2010), are forced to produce bifurcating trees. Therefore, we here only consider the site patterns xxyy, xyxy, xyyx that can produce a bifurcating tree. The probability of xxyy is given by

(5) $$\begin{array}{l} \frac{12}{4}\left(P_{AA}\left(t_{1}\right)P_{AA}\left(t_{2}\right)P_{AC}\left(t_{3}\right)P_{AC}\left(t_{4}\right)\right.\\ \;+P_{CA}\left(t_{1}\right)P_{CA}\left(t_{2}\right)P_{CC}\left(t_{3}\right)P_{CC}\left(t_{4}\right)\\ \;+P_{GA}\left(t_{1}\right)P_{GA}\left(t_{2}\right)P_{GC}\left(t_{3}\right)P_{GC}\left(t_{4}\right)\\ \;\left.+P_{TA}\left(t_{1}\right)P_{TA}\left(t_{2}\right)P_{TC}\left(t_{3}\right)P_{TC}\left(t_{4}\right)\right) \end{array}$$

The probability of xyxy is given by

(6) $$\begin{array}{l} \frac{12}{4}\left(P_{AA}\left(t_{1}\right)P_{AC}\left(t_{2}\right)P_{AA}\left(t_{3}\right)P_{AC}\left({\text{t}}_{4}\right)\right.\\ \;+P_{CA}\left(t_{1}\right)P_{CC}\left(t_{2}\right)P_{CA}\left(t_{3}\right)P_{CC}\left(t_{4}\right)\\ \;+P_{GA}\left(t_{1}\right)P_{GC}\left(t_{2}\right)P_{GA}\left(t_{3}\right)P_{GC}\left(t_{4}\right)\\ \;\left.+P_{TA}\left(t_{1}\right)P_{TC}\left(t_{2}\right)P_{TA}\left(t_{3}\right)P_{TC}\left(t_{4}\right)\right) \end{array}$$

Similarly, the probability of xyyx is given by

(7) $$\begin{array}{l} \frac{12}{4}\left(P_{AA}\left(t_{1}\right)P_{AC}\left(t_{2}\right)P_{AC}\left(t_{3}\right)P_{AA}\left(t_{4}\right)\right.\\ \;+P_{CA}\left(t_{1}\right)P_{CC}\left(t_{2}\right)P_{CC}\left(t_{3}\right)P_{CA}\left(t_{4}\right)\\ \;+P_{GA}\left(t_{1}\right){\text{P}}_{GC}\left(t_{2}\right)P_{GC}\left(t_{3}\right)P_{GA}\left(t_{4}\right)\\ \;\left.+P_{TA}\left(t_{1}\right)P_{TC}\left(t_{2}\right)P_{TC}\left(t_{3}\right)P_{TA}\left(t_{4}\right)\right) \end{array}$$

If $t_{1}=t_{2}=t_{3}=t_{4}$, then $P\left(xxyy\right)=P\left(xyxy\right)=P\left(xyyx\right)$. The ML tree is an unbiased estimator of the 4-taxon star tree $T^{\text{*}}$ if $\;P\left({\hat{T}}_{MLE}=T_{1}\right)=P\left({\hat{T}}_{MLE}=T_{2}\right)=P\left({\hat{T}}_{MLE}=T_{3}\right)$. If the sequence length K=1, the probability $P\left({\hat{T}}_{MLE}=T_{1}\right)$ is equal to the probability $P\left(xxyy\right)$, and $P\left({\hat{T}}_{MLE}=T_{2}\right)=P\left(xyxy\right)$, and $P\left({\hat{T}}_{MLE}=T_{3}\right)=P\left(xyyx\right)$. Thus, the ML tree ${\hat{T}}_{MLE}$ is an unbiased estimator if the 4-taxon star tree $T^{\text{*}}$ has equal branch lengths $t_{1}=t_{2}=t_{3}=t_{4}$. If the branch lengths are not equal, for example, $t_{1}<t_{2}<t_{3}<t_{4}$, then $P\left(xxyy\right)>P\left(xyxy\right)>P\left(xyyx\right)$. It follows that $P\left({\hat{T}}_{MLE}=T_{1}\right)>P\left({\hat{T}}_{MLE}=T_{2}\right)$ and $P\left({\hat{T}}_{MLE}=T_{1}\right)>P\left({\hat{T}}_{MLE}=T_{3}\right)$. Thus, the ML tree ${\hat{T}}_{MLE}$ is a biased estimator of the 4-taxon star tree $T^{\text{*}}$ with unequal branch lengths. Moreover, the ML tree ${\hat{T}}_{MLE}$ favors the tree $T_{1}$ which groups the lineages of ${\text{S}}_{1}$ and ${\text{S}}_{2}$ with short branches $t_{1}$ and $t_{2}$. We call this phenomenon short branch attraction (SBA).

If the sequence length K is large, there is at least one site with the pattern xxyy, xyxy, or xyyx in the sequence alignment D. As a result, ML methods would consistently produce a bifurcating tree as the estimate of the 4-taxon star tree $T^{\text{*}}$. Let $\lambda =P\left(xxyy\right)+P\left(xyxy\right)+P\left(xyyx\right)$. The probability that the sequence alignment of length K consists of at least one site with the patterns xxyy, xyxy, and xyyx is $1-{\left(1-\lambda \right)}^{K}$, which converges to 1.0 as K goes to infinity. Note that the probability that the counts of three patterns are equal, the case that ML methods estimate a star tree, converges to 0 as K→∞. It indicates that the MLE $T_{MLE}$ converges to a bifurcating tree as the sequence length K goes to infinity. Thus, $T_{MLE}$ is a biased estimator of the star tree $T^{\text{*}}$ if the sequence length K is large. Moreover, ML methods favor the tree $T_{1}=\left(\left(S_{1},S_{2}\right),S_{3},S_{4}\right)$ over the other two trees $T_{2}=\left(\left(S_{1},S_{3}\right),S_{2},S_{4}\right)$ and $T_{3}=\left(\left(S_{1},S_{4}\right),S_{2},S_{3}\right)$ if the count of the pattern xxyy outnumbers the counts of the patterns xyxy and xyyx, or equivalently, $P\left(xxyy\right)>P\left(xyxy\right)$ and $P\left(xxyy\right)>P\left(xyyx\right)$.

It can be shown that if $t_{1}$, $t_{2}<t_{3}$, $t_{4}$, then $P\left(xxyy\right)>P\left(xyxy\right)$ and $P\left(xxyy\right)>P\left(xyyx\right)$ (Supplementary Appendix A2). This result indicates that the MLE ${\hat{T}}_{MLE}$ of the 4-taxon star tree $T^{\text{*}}$ is biased toward the bifurcating tree $T_{1}$ which groups the lineages of $S_{1}$ and $S_{2}$ due to SBA. The probability $P\left({\hat{T}}_{MLE}=T_{1}\right)$, or equivalently the biasness of ${\hat{T}}_{MLE}$, increases as the branch lengths $t_{1}$ and $t_{2}$ decrease and/or $t_{3}$ and $t_{4}$ increase.

##### 2.1.1.2. Scenario 2: 4-taxon bifurcating trees

In this section, the true tree $T^{\text{*}}$ is assumed to be a 4-taxon bifurcating tree $T^{\text{*}}=T_{2}=\left(\left(S_{1}:t_{1},S_{3}:t_{3}\right):t_{0},S_{2}:t_{2},S_{4}:t_{4}\right)$ with an internal branch $\left(\text{length}=T_{0}\right)$ and four terminal branches $\left(\text{length}=t_{1},t_{2},t_{3},t_{4}\right)$ (Figure 1B). To calculate the probabilities of 15 site patterns, we consider two scenarios – the nucleotides $x_{n_{1}}$ and $x_{n_{2}}$ at two internal nodes $n_{1}$ and $n_{2}$ are identical or distinct. Under the Jukes-Cantor model, the probability of two identical nucleotides is $P\left(x_{n_{1}}=x_{n_{2}}|t_{0}\right)=\frac{1}{4}+\frac{3}{4}e^{-4t_{0}/3}$, while the probability of two distinct nucleotides is $P\left(x_{n_{1}}\neq x_{n_{2}}|t_{0}\right)=1-P\left(x_{n_{1}}=x_{n_{2}}|t_{0}\right)=\frac{3}{4}-\frac{3}{4}e^{-4t_{0}/3}$. If the nucleotides $x_{n_{1}}$ and $x_{n_{2}}$ at two internal nodes are identical, the probability of a site pattern &=WXYZ coincides with that for a star tree described in equation (3),

(8) $$P\left(\&=WXYZ|T^{\text{*}},t_{1},t_{2},t_{3},t_{4},x_{n_{1}}=x_{n_{2}}\right)\;=\frac{1}{4}\sum_{H=A,C,G,T}P_{HW}\left(t_{1}\right)P_{HX}\left(t_{2}\right)P_{HY}\left(t_{3}\right)P_{HZ}\left(t_{4}\right)$$

If two nucleotides $x_{n_{1}}$ and $x_{n_{2}}$ are distinct, the probability of the site pattern & is given by

(9) $$P\left(\&=WXYZ|T^{\text{*}},t_{1},t_{2},t_{3},t_{4},x_{n_{1}}\neq x_{n_{2}}\right)\;=\frac{1}{16}\sum_{H=A,C,G,T}\sum_{L\neq H}P_{HW}\left(t_{1}\right)P_{HX}\left(t_{2}\right)P_{LY}\left(t_{3}\right)P_{LZ}\left(t_{4}\right)$$

The probability of the site pattern & is equal to the weighted sum of the two probabilities in equations (7) and (8), i.e.,

(10) $$\begin{array}{l} P\left(\&|T^{\text{*}},t_{0},t_{1},t_{2},t_{3},t_{4}\right)\\ =P\left(\&|T^{\text{*}},t_{1},t_{2},t_{3},t_{4},x_{n_{1}}=x_{n_{2}}\right)P\left(x_{n_{1}}=x_{n_{2}}|t_{0}\right)\\ +P\left(\&|T^{\text{*}},t_{1},t_{2},t_{3},t_{4},x_{n_{1}}\neq x_{n_{2}}\right)P\left(x_{n_{1}}\neq x_{n_{2}}|t_{0}\right) \end{array}$$

As $t_{0}\to 0$, $P\left(x_{n_{1}}\neq x_{n_{2}}|t_{0}\right)\to 0$ and $P\left(x_{n_{1}}=x_{n_{2}}|t_{0}\right)\to 1$. The probability of the site pattern & converges to the probability $P\left(\&|T^{\text{*}},t_{1},t_{2},t_{3},t_{4},x_{n_{1}}=x_{n_{2}}\right)$ derived from 4-taxon star trees. Thus, when the internal branch length $t_{0}$ is small, according to the theory derived for 4-taxon star trees in the previous section, the ML tree is a biased estimator of $T^{\text{*}}=T_{2}$, favoring the wrong tree $T_{1}$ due to SBA. As $t_{0}\to \infty$, $P\left(x_{n_{1}}\neq x_{n_{2}}|t_{0}\right)\to 1$ and $P\left(x_{n_{1}}=x_{n_{2}}|t_{0}\right)\to 0$, and the probability of the site pattern & converges to the probability $P\left(\&|T^{\text{*}},t_{1},t_{2},t_{3},t_{4},x_{n_{1}}\neq x_{n_{2}}\right)$. Moreover, if the nucleotides at two internal nodes are distinct, the probability $P\left(\&=xyxy|T^{\text{*}},t_{1},t_{2},t_{3},t_{4},x_{n_{1}}\neq x_{n_{2}}\right)$ of the site pattern xxyy is great than the probability $P\left(\&=xxyy|T^{\text{*}},t_{1},t_{2},t_{3},t_{4},x_{n_{1}}\neq x_{n_{2}}\right)$ and the probability $\;P\left(\&=xyyx|T^{\text{*}},t_{1},t_{2},t_{3},t_{4},x_{n_{1}}\neq x_{n_{2}}\right)$ (Supplementary Appendix A3). It indicates that the site patterns generated from $P\left(\&|T^{\text{*}},t_{1},t_{2},t_{3},t_{4},x_{n_{1}}\neq x_{n_{2}}\right)$ always support the true tree $T^{\text{*}}$ (Figure 1B). Thus, as the internal branch length $t_{0}\to \infty$, the MLE ${\hat{T}}_{MLE}$ becomes an unbiased estimator of $T^{\text{*}}$.

##### 2.1.1.3. Scenario 3: generalization to the trees of more than 4 taxa

Let T be an n-taxon $\left(n>4\right)$ bifurcating tree (Figure 1C). Let $n_{01}$ and $n_{02}$ be the two nodes at the two ends of an internal branch b of T. Because T is a bifurcating tree, each of the two internal nodes $n_{01}$ and $n_{02}$ is also associated with two other nodes (Figure 1C). Let $n_{1}$ and $n_{3}$ be the two nodes associated with $n_{01}$. Let $n_{2}$ and $n_{4}$ be the two nodes associated with $n_{02}$ (Figure 1C). The nodes $n_{1}$ and $n_{2}$ are terminal nodes, while the nodes $n_{3}$ and $n_{4}$ could be the terminal or internal nodes in the n-taxon tree T. The six nodes along with the five branches connecting them form a 4-taxon subtree $T^{\text{*}}=\left(\left(n_{1},n_{3}\right),n_{2},n_{4}\right)$ (i.e., the subtree in brown, Figure 1C), where b is the “internal” branch of length $t_{0}$ and the remaining four are “terminal” branches of length $t_{1}<t_{2}<t_{3}<t_{4}$. The theory derived for Scenario 1 and 2 indicates that there are 15 patterns with distinct probabilities for the nucleotides at the four “terminal” nodes $n_{1}$, $n_{2}$, $n_{3}$, $n_{4}$. Here, we ask the same question: what are the probabilities of the 15 site patterns? Firstly, those probabilities do not depend on the other parts of the n-taxon tree T (i.e., the blue subtrees in Figure 1C). Secondly, if the assumed substitution model is time reversible – in fact, most substitution models are time reversible, then the theory derived in Scenario 2 can be applied to computing the probabilities of 15 site patterns for this 4-taxon subtree $T^{\text{*}}$. Specifically, the probability of the site pattern & is equal to the sum of the probabilities of the site pattern & when the nucleotides $x_{n_{01}}$ and $x_{n_{02}}$ at two internal nodes $n_{01}$ and $n_{02}$ are identical or distinct as described in equation (9). Finally, according to the theory derived for Scenario 2, if the internal branch length $t_{0}$ is small (i.e., $t_{0}\to 0$), the ML tree $T_{MLE}$ is a biased estimator of $T^{\text{*}}=\left(\left(n_{1},n_{3}\right),n_{2},n_{4}\right)$, favoring the wrong tree $\left(\left(n_{1},n_{2}\right),n_{3},n_{4}\right)$ due to SBA. However, because the nucleotides at the nodes $n_{3}$, $n_{4}$ are not observable, it is difficult to formally prove that the ML tree ${\hat{T}}_{MLE}$ is a biased estimator of $T^{\text{*}}$ when $t_{0}$ is small and the branch lengths $t_{1}$ and $t_{2}$ are much less than the branch lengths $t_{3}$ and $t_{4}$. Instead, the biased MLE of the subtree $T^{\text{*}}$ will be illustrated by simulation.

#### 2.1.2. Short branch attraction in species trees

In this section, we analyze three distinct scenarios (4-taxon star trees, 4-taxon bifurcating trees, and n-taxon trees) for species trees. We demonstrate that the short branch attraction artifact has the potential to lead to erroneous species tree estimation in these scenarios

##### 2.1.2.1. Scenario 1: 4-taxon star species trees

Let $S^{\text{*}}=\left(S_{1}:\tau_{1},S_{2}:\tau_{2},S_{3}:\tau_{3},S_{4}:\tau_{4}\right)$ be a 4-taxon star species tree with unequal branch lengths $\tau_{1}<\tau_{2}<\tau_{3}<\tau_{4}$ (Figure 1D). Let $\theta_{0}$ be the population size parameter of the ancestral population at the root of the species tree $S^{\text{*}}$. It is assumed that a single allele is sampled from each species. The gene tree $T^{\text{*}}$ generated from this star species tree under the multispecies coalescent model is a 4-taxon bifurcating tree with unequal branch lengths. Under the coalescent model, the three unrooted gene trees $T_{1}=\left(\left(S_{1},S_{2}\right),S_{3},S_{4}\right)$, $T_{2}=\left(\left(S_{1},S_{3}\right),S_{2},S_{4}\right)$, and $T_{3}=\left(\left(S_{1},S_{4}\right),S_{2},S_{3}\right)$ have the same probability $\left(\frac{1}{3},\frac{1}{3},\frac{1}{3}\right)$. Let $t_{0}$ be the length of the internal branch of $T^{\text{*}}$. It follows from the coalescent theory that the internal branch length $t_{0}$ has the exponential distribution with mean $\frac{5}{9}\theta$. When θ is small (i.e., θ→0), most gene trees generated from the star species tree $S^{\text{*}}$ have a short internal branch and four terminal branches whose lengths $t_{1}<t_{2}<t_{3}<t_{4}$ tend to be in the same order as those $\tau_{1}<\tau_{2}<\tau_{3}<\tau_{4}$ in the species tree $S^{\text{*}}$ Due to SBA, the MLEs of such 4-taxon gene trees support the tree $T_{1}=\left(\left(S_{1},S_{2}\right),S_{3},S_{4}\right)$ with a probability ⊕1.0, which significantly deviates from the true probability distribution $\left(\frac{1}{3},\frac{1}{3},\frac{1}{3}\right)$ of the gene trees derived from the species tree $S^{\text{*}}$ under the multispecies coalescent model. As a result, the biased gene trees mislead the coalescent methods to consistently estimate the wrong species tree $\left(\left(S_{1},S_{2}\right),S_{3},S_{4}\right)$ as the number of gene trees goes to infinity. Thus, SBA occurs in the 4-taxon star species trees.

##### 2.1.2.2. Scenario 2: 4-taxon bifurcating species trees

Consider a 4-taxon bifurcating species tree $S^{\text{*}}=\left(\left(S_{1}:\tau_{1},S_{3}:\tau_{3}\right),S_{2}:\tau_{2},S_{4}:\tau_{4}\right)$ (Figure 1E). Let $\tau_{1}$ and $\tau_{2}$ be the branch lengths and $\theta_{1}$ and $\theta_{2}$ be the population size parameters of two internal branches (Figure 1E). The probability that two alleles from species $S_{1}$ and $S_{2}$ do not coalesce in their most recent common ancestral population (MRCA) is $p_{1}=1-\frac{1}{\theta_{1}}e^{-\frac{\tau_{1}}{\theta_{1}}}$. The probability that three alleles from species $S_{1}$, $S_{2}$, and $S_{3}$ do not coalesce in their MRCA $p_{2}=1-\frac{3}{\theta_{2}}e^{-\frac{3\tau_{2}}{\theta_{2}}}$. The probability p that the four alleles coalesce in the root population is equal to the probability $p_{1}$ that two alleles from species $S_{1}$ and $S_{2}$ do not coalesce in their MRCA multiplied by the probability $p_{2}$ that three alleles from species $S_{1}$, $S_{2}$, and $S_{3}$ do not coalesce in their MRCA, i.e., $p=p_{1}p_{2}=\left(1-\frac{1}{\theta_{1}}e^{-\frac{\tau_{1}}{\theta_{1}}}\right)\left(1-\frac{3}{\theta_{2}}e^{-\frac{3\tau_{2}}{\theta_{2}}}\right)$. If two ratios $\frac{\tau_{1}}{\theta_{1}}$ and $\frac{\tau_{2}}{\theta_{2}}$ are small (i.e., $\theta_{1}$ and $\theta_{2}$ are large, or $\tau_{1}$ and $\tau_{2}$ are small), the four alleles from species $S_{1}$, $S_{2}$, $S_{3}$, and $S_{4}$ have a high probability of coalescing in the root population, a case described in Scenario 1: 4-taxon star species trees for which the coalescent methods consistently estimate the wrong species tree $\left(\left(S_{1},S_{2}\right),S_{3},S_{4}\right)$. Thus, SBA occurs in the 4-taxon bifurcating species trees if the population size parameters $\theta_{1}$ and $\theta_{2}$ of the two ancestral populations are large, or the branch length $\tau_{1}$ and $\tau_{2}$ are small.

##### 2.1.2.3. Scenario 3: n-taxon bifurcating species trees

Let $S^{\text{*}}$ be the 4-taxon subtree (i.e., the subtree in red, Figure 1F) of an n-taxon $\left(n>4\right)$ bifurcating species tree S. Let $n_{1}$, $n_{2}$, $n_{3}$, $n_{4}$ be the four terminal nodes of $S^{\text{*}}$ (Figure 1F). Note that the nodes $n_{3}$ and $n_{4}$ are internal nodes, while the nodes $n_{1}$ and $n_{2}$ are two terminal nodes in the n-taxon species tree S. It is assumed that one allele is sampled from each species. If all genealogical lineages coalesce in the blue subtrees below the nodes $n_{3}$, $n_{4}$ (Figure 1F), then only one lineage enters each of the nodes $n_{3}$, $n_{4}$. In this case, the coalescence process occurring in the subtree $S^{\text{*}}$ is the same process as that occurs in the 4-taxon species tree described in the previous section Scenario 2: 4 -taxon bifurcating species trees. Thus, SBA may occur in the 4-taxon subtree $S^{\text{*}}$ of an n-taxon species tree if the population size parameter $\theta_{0}$ in the root population is small, and the population size parameters $\theta_{1}$ and $\theta_{2}$ of the two ancestral populations are large, or the branch length $\tau_{1}$ and $\tau_{2}$ are small. If multiple lineages enter the nodes $n_{3}$, $n_{4}$, the gene tree lineages generated from the subtree $S^{\text{*}}$ involve more than 4 taxa. Although SBA may still occur in the n-taxon gene trees (see Scenario 3: Generalization to the trees of more than 4 taxa) which can subsequently mislead the coalescent methods to estimate the wrong species tree, it is difficult to formally prove that SBA can occur in the n-taxon species trees. Instead, we will use simulation to illustrate SBA in the species trees of more than 4 species.

### 2.2. Simulation study

#### 2.2.1. The algorithmic bias of PhyML and RAxML

This section is to assess the algorithmic bias of two popular phylogenetic programs, PhyML and RAxML, in building the ML trees from sequence alignments. Later, when we use simulation to illustrate that ML trees are biased estimators, we need to show that the level of the biasness of ML trees exceeds what the algorithmic bias can explain, i.e., the biasness of ML trees is due to model or data deficiencies rather than some unnoticed “bugs” in the computational algorithms implemented in PhyML and RAxML.

##### 2.2.1.1. Scenario 1: identical sequences

**FIGURE 2:**
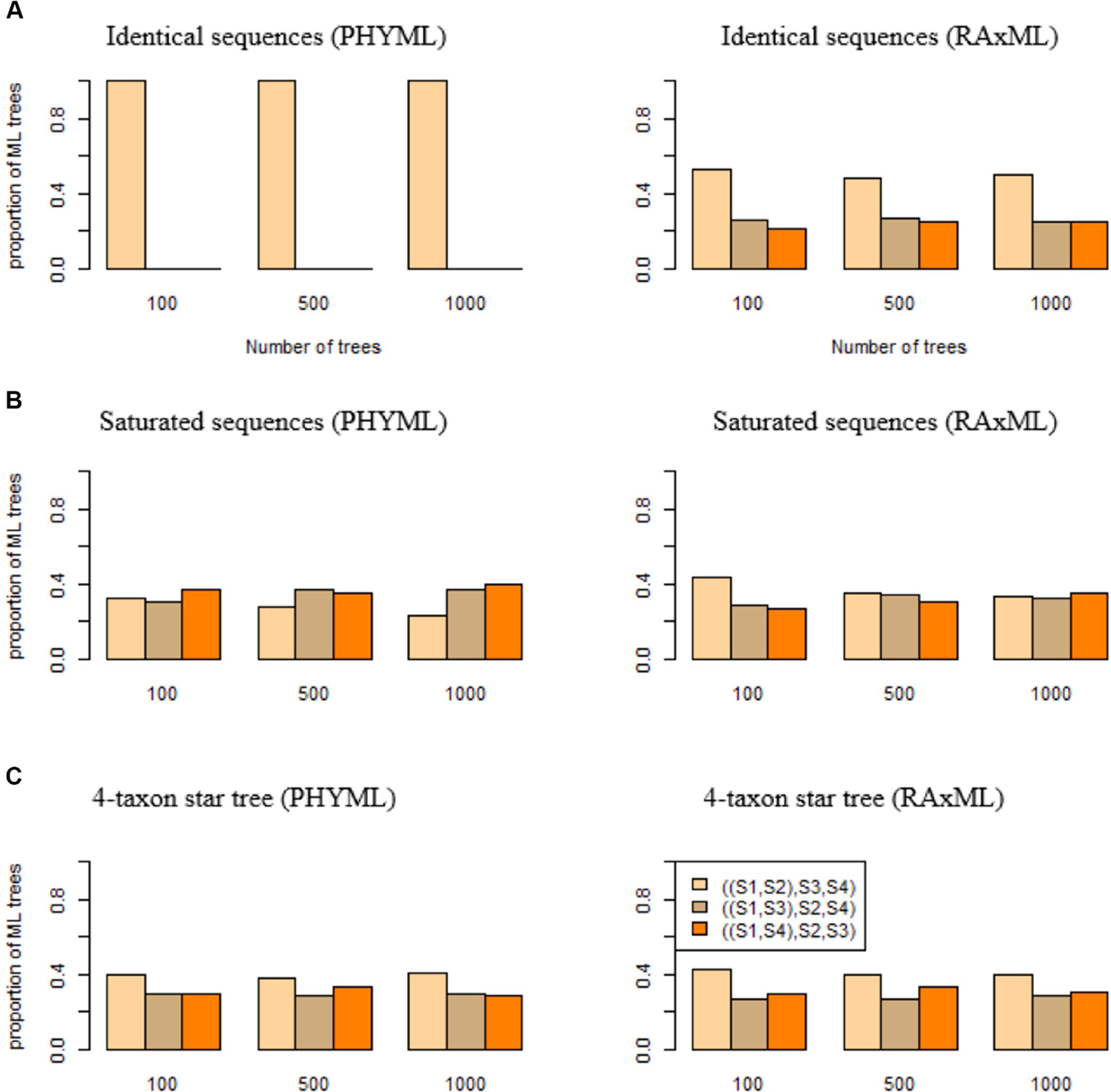
The algorithmic bias of PhyML and RAxML. ML trees were built by PhyML (left panel) and RAxML (right panel) for **(A)** identical sequences, **(B)** saturated sequences, and **(C)** sequences generated from a 4-taxon star tree (${\text{S}}_{1}:0.01$, ${\text{S}}_{2}:0.01$, ${\text{S}}_{3}:0.01$, ${\text{S}}_{4}:0.01$). The proportions of three ML trees $\left(\left(S_{1},S_{2}\right),S_{3},S_{4}\right)$, $\left(\left(S_{1},S_{3}\right),S_{2},S_{4}\right)$, and $\left(\left(S_{1},S_{4}\right),S_{2},S_{3}\right)$ were calculated and compared with the expected proportions $\left(1/3,1/3,1/3\right)$ if two phylogenetic programs PhyML and RAxML do not have an algorithmic bias.

If DNA sequences are identical across four species $S_{1}$, $S_{2}$, $S_{3}$, $S_{4}$, phylogenetic programs are expected to estimate three bifurcating trees $\left(\left(S_{1},S_{2}\right),S_{3},S_{4}\right)$, $\left(\left(S_{1},S_{3}\right),S_{2},S_{4}\right)$, and $\left(\left(S_{1},S_{4}\right),S_{2},S_{3}\right)$ with equal probabilities $\left(\frac{1}{3},\frac{1}{3},\frac{1}{3}\right)$. Deviation from this uniform probability distribution indicates an algorithmic bias in phylogenetic programs. Here, we consider two popular maximum likelihood (ML) phylogenetic programs PhyML and RAxML. Identical sequences of 1,000 base pairs (bps) were generated for species $S_{1}$, $S_{2}$, $S_{3}$, $S_{4}$. Surprisingly, 100% of the ML trees estimated from identical sequences by PhyML are $\left(\left(S_{1},S_{2}\right),S_{3},S_{4}\right)$ (Figure 2A), which is consistent with the previous results that PhyML tends to infer one particular bifurcating topology even though the true relationship is a polytomy (Xi et al., 2015). By contrast, the proportions of three ML trees reconstructed by RAxML are close to the expected probabilities $\left(\frac{1}{3},\frac{1}{3},\frac{1}{3}\right)$. For the replicates of 100,500, and 1,000 trees, the proportion of $\left(\left(S_{1},S_{2}\right),S_{3},S_{4}\right)$ estimated by RAxML is 0.53, 0.48, 0.5, respectively (Figure 2A), which is significantly higher than the expected probability $\frac{1}{3}$.

##### 2.2.1.2. Scenario 2: saturated sequences

DNA sequences are saturated if the nucleotides in the sequences are identically and independently distributed among species with the limiting probability distribution derived from the substitution model. Saturated sequences may arise when a phylogenetic tree has long terminal branches. If sequences are saturated across species $S_{1}$, $S_{2}$, $S_{3}$, $S_{4}$, phylogenetic programs are expected to produce three bifurcating trees $\left(\left(S_{1},S_{2}\right),S_{3},S_{4}\right)$, $\left(\left(S_{1},S_{3}\right),S_{2},S_{4}\right)$, and $\left(\left(S_{1},S_{4}\right),S_{2},S_{3}\right)$ with equal probability $\left(\frac{1}{3},\frac{1}{3},\frac{1}{3}\right)$. Saturated sequences of 1000 bpS were simulated for species $S_{1}$, $S_{2}$, $S_{3}$, $S_{4}$ and then used to build phylogenetic trees. The results indicate that an algorithmic bias of PhyML and RAxML for saturated sequences is less severe than that for identical sequences (Figure 2B). PhyML appears to favor the tree $\left(\left(S_{1},S_{4}\right),S_{2},S_{3}\right)$, as 40% of the ML trees reconstructed by PhyML are $\left(\left(S_{1},S_{4}\right),S_{2},S_{3}\right)$ (Figure 2B), which is significantly higher than the expected proportion 1/3. By contrast, RAxML appears to favor the tree $\left(\left(S_{1},S_{2}\right),S_{3},S_{4}\right)$ when the number of trees is 100 or 500, but the proportion of $\left(\left(S_{1},S_{2}\right),S_{3},S_{4}\right)$ is 0.33 when the number of trees is 1,000 (Figure 2B), which is not significantly different from the expected proportion 1/3.

##### 2.2.1.3. Scenario 3: sequences from a 4-taxon star tree

Identical or saturated sequences are not frequently observed in real sequence data. Here, we consider a more realistic scenario where the true phylogenetic tree is a 4-taxon star tree. In this simulation, DNA sequences were generated from the tree ($S_{1}:0.01$, $S_{2}:0.01$, $S_{3}:0.01$, $S_{4}:0.01$) with equal branch lengths of 0.01. When the true tree is a polytomy tree, three ML trees $\left(\left(S_{1},S_{2}\right),S_{3},S_{4}\right)$, $\left(\left(S_{1},S_{3}\right),S_{2},S_{4}\right)$, and $\left(\left(S_{1},S_{4}\right),S_{2},S_{3}\right)$ are expected to be uniformly distributed with equal probabilities $\left(1/3,1/3,1/3\right)$. Deviation from this uniform distribution indicates an algorithmic bias of PhyML and RAxML. The simulation results suggest that both PhyML and RAxML favor the tree $\left(\left(S_{1},S_{2}\right),S_{3},S_{4}\right)$. When the number of trees is 1,000, the proportion of the tree $\left(\left(S_{1},S_{2}\right),S_{3},S_{4}\right)$ estimated by PhyML and RAxML is 0.41 and 0.4, respectively (Figure 2C), significantly higher than the expected proportion 1/3.

The three simulations (identical sequences, saturated sequences, and 4-taxon star tree) indicate that both programs (PHYML and RAxML) suffer an algorithmic bias, but the algorithmic bias of PHYML appears to be more severe than that of RAxML. Therefore, we will use RAxML to build ML trees in the subsequent analyses.

#### 2.2.2. Short branch attraction in gene trees

##### 2.2.2.1. Scenario 1: 4-taxon star trees with unequal branch lengths

**FIGURE 3:**
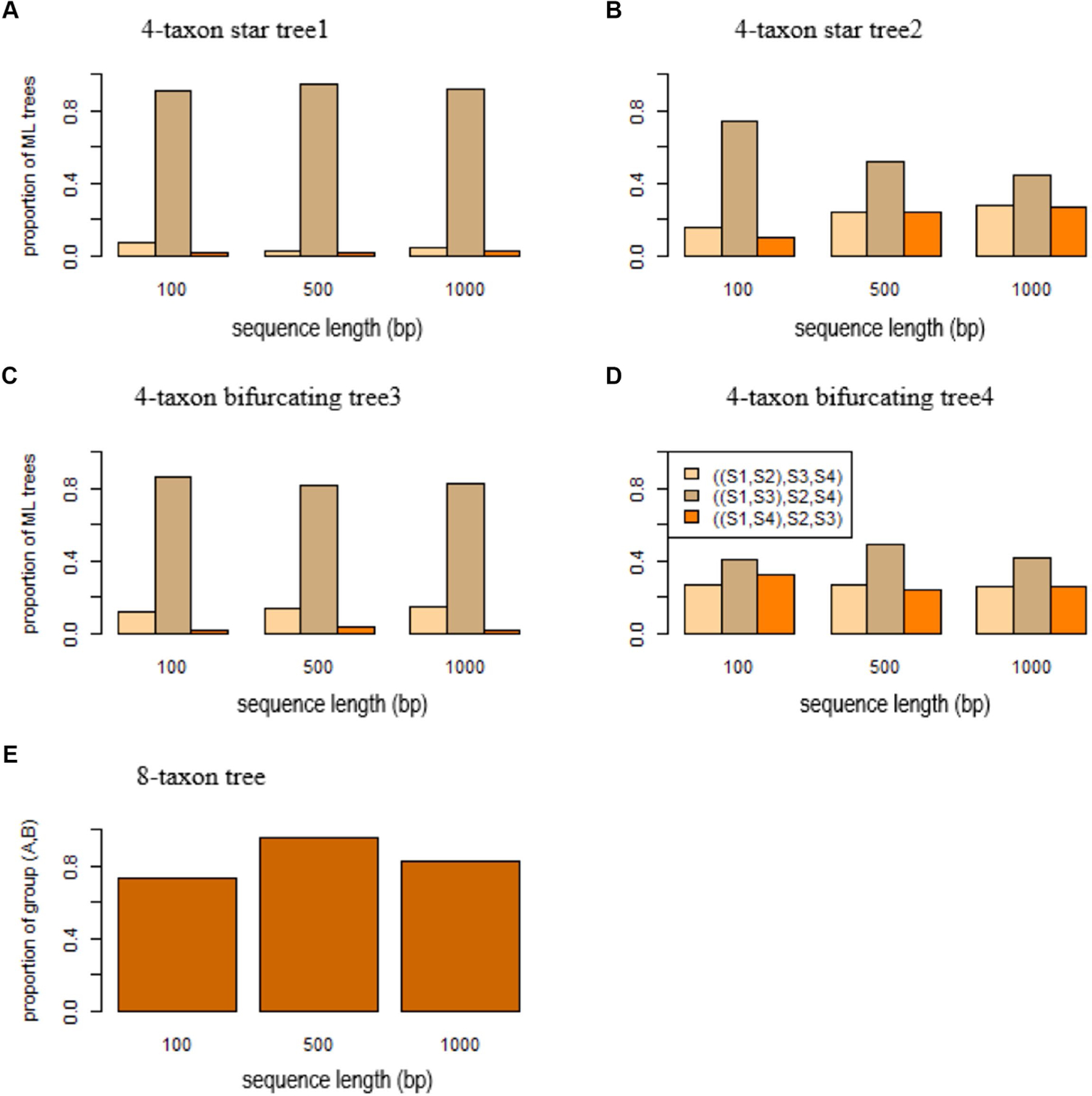
Short branch attraction in gene trees. ML trees were built by RAxML for the sequences simulated from **(A)** the 4-taxon star tree1 (${\text{S}}_{1}:0.0001$, ${\text{S}}_{2}:0.01$, ${\text{S}}_{3}:0.0001$, ${\text{S}}_{4}:0.01$), **(B)** the 4-taxon tree2 (${\text{S}}_{1}:0.1$, ${\text{S}}_{2}:0.01$, ${\text{S}}_{3}:0.1$, ${\text{S}}_{4}:0.01$), **(C)** the 4-taxon bifurcating tree3 ($\left({\text{S}}_{1}:0.0001,{\text{S}}_{2}:0.01\right):0.0001$, ${\text{S}}_{3}:0.0001$, ${\text{S}}_{4}:0.01$), **(D)** the 4-taxon bifurcating tree4 ($\left({\text{S}}_{1}:0.1,{\text{S}}_{2}:0.01\right):0.0001$, ${\text{S}}_{3}:0.1$, ${\text{S}}_{4}:0.01$), and **(E)** the 8-taxon tree (A: 0.0001, ($\left({\text{S}}_{1}:0.01,{\text{S}}_{2}:0.01\right):0.01$, ${\text{S}}_{3}:0.01$): 0.01:0.0001, B: 0.0001, ($\left({\text{S}}_{4}:0.01,{\text{S}}_{5}:0.01\right):0.01$, ${\text{S}}_{6}:0.0$):0.01) with two short branches $\left(\text{length}=0.0001\right)$ leading to the species A and B. The y-axis in **(A-D)** is the proportion of three ML trees $\left(\left({\text{S}}_{1},{\text{S}}_{2}\right),{\text{S}}_{3},{\text{S}}_{4}\right)$, $\left(\left({\text{S}}_{1},{\text{S}}_{3}\right),{\text{S}}_{2},{\text{S}}_{4}\right)$, and $\left(\left({\text{S}}_{1},{\text{S}}_{4}\right),{\text{S}}_{2},{\text{S}}_{3}\right)$, while the y-axis in **(E)** is the proportion of ML trees with the group **(A,C)**. The x-axis is the sequence length 100,500, and 1,000 bp

According to the theory we developed above, the MLE of a 4 -taxon star tree $T^{\text{*}}$ with unequal branch lengths favors a bifurcating tree in which two lineages with short branches are grouped together. Here, we use simulation to demonstrate the biasness of ML methods in estimating 4-taxon star trees with unequal branch lengths. In this simulation, the 4-taxon star tree ($S_{1}:0.0001$, $S_{2}:0.01$, $S_{3}:0.0001$, $S_{4}:0.01$) has two short branches of length 0.0001 leading to species $S_{1}$ and $S_{3}$. DNA sequences were simulated from this 4-taxon star tree and then used to build ML trees by RAxML. The simulation was repeated 100 times and we calculated the proportions of three ML trees $\left(\left(S_{1},S_{2}\right),S_{3},S_{4}\right)$, $\left(\left(S_{1},S_{3}\right),S_{2},S_{4}\right)$, and $\left(\left(S_{1},S_{4}\right),S_{2},S_{3}\right)$. As expected, more than 90% of the ML trees are $\left(\left(S_{1},S_{3}\right),S_{2},S_{4}\right)$ (Figure 3A). When the true species tree is ($S_{1}:0.1$, $S_{2}:0.01$, $S_{3}:0.1$, $S_{4}:0.01$) in which the length of two short terminal branches increases from 0.0001 to 0.1, most of the ML trees are still $\left(\left(S_{1},S_{3}\right),S_{2},S_{4}\right)$, but the probability of the most probable ML tree drops from 0.91 to 0.74 for sequence length=100 bps (Figure 3B). As the sequence length increases, the probability drops from 0.75 to 0.45 (Figure 3B).

##### 2.2.2.2. Scenario 2: 4-taxon bifurcating trees with unequal branch lengths

Likewise, SBA occurs in the 4 -taxon bifurcating trees, misleading ML methods to produce the wrong tree estimates. To assess the effects of SBA in gene trees, DNA sequences of 100,500, and 1,000 bps were simulated from a 4 -taxon bifurcating tree ($\left(S_{1}:0.0001,S_{2}:0.01\right):0.0001$, $S_{3}:0.0001$, $S_{4}:0.01$). Because the terminal branches leading to the species $S_{1}$ and $S_{3}$ are short, the sequences of species $S_{1}$ and $S_{3}$ generated from this tree are almost identical to each other. As a result, the ML methods group the species $S_{1}$ and $S_{3}$ and estimate the wrong tree $\left(\left(S_{1},S_{3}\right),S_{2},S_{4}\right)$ (Figure 3C). The simulation results suggest that 80% of the ML trees reconstructed by RAxML are the tree $\left(\left(S_{1},S_{3}\right),S_{2},S_{4}\right)$ (Figure 3C). When the true species tree is ($\left(S_{1}:0.01,S_{2}:0.01\right):0.0001$, $S_{3}:0.01$, $S_{4}:0.01$) in which the length of two short terminal branches increases from 0.0001 to 0.1, most of the ML trees are still $\left(\left(S_{1},S_{3}\right),S_{2},S_{4}\right)$, but the probability of the most probable ML tree drops from 0.86 to 0.41 for sequence length=100 bps (Figure 3D). As the sequence length increases, the probability increases from 0.41 for sequence length=100 bps to 0.49 for 500 bps and 0.42 for 1,000 (Figure 3D)

##### 2.2.2.3. Scenario 3: SBA in n-taxon trees

DNA sequences were simulated from the 8-taxon tree ((A: 0.0001, ($\left({\text{S}}_{1}:0.01,{\text{S}}_{2}:0.01\right):0.01$, ${\text{S}}_{3}:0.01$): 0.01): 0.0001, B: 0.0001, ($\left({\text{S}}_{4}:0.01,{\text{S}}_{5}:0.01\right):0.01$, ${\text{S}}_{6}:0.01$): 0.01) with two short branches $\left(\text{length}=0.0001\right)$ leading to the species A and B. The species A and B are placed in two monophyletic clades – $\left(\text{A},{\text{S}}_{1},{\text{S}}_{2},{\text{S}}_{3}\right)$ and $\left(\text{B},{\text{S}}_{4},{\text{S}}_{5},{\text{S}}_{6}\right)$ of the 8-taxon tree. However, when the sequence length is 100 bps, the species A and B are mistakenly grouped together in 73% of the ML trees reconstructed by RAxML (Figure 3E). The percentage increases to 96 and 83%, respectively, for the sequence length=500 and 1,000 bps (Figure 3E).

#### 2.2.3. Short branch attraction in species trees

**FIGURE 4:**
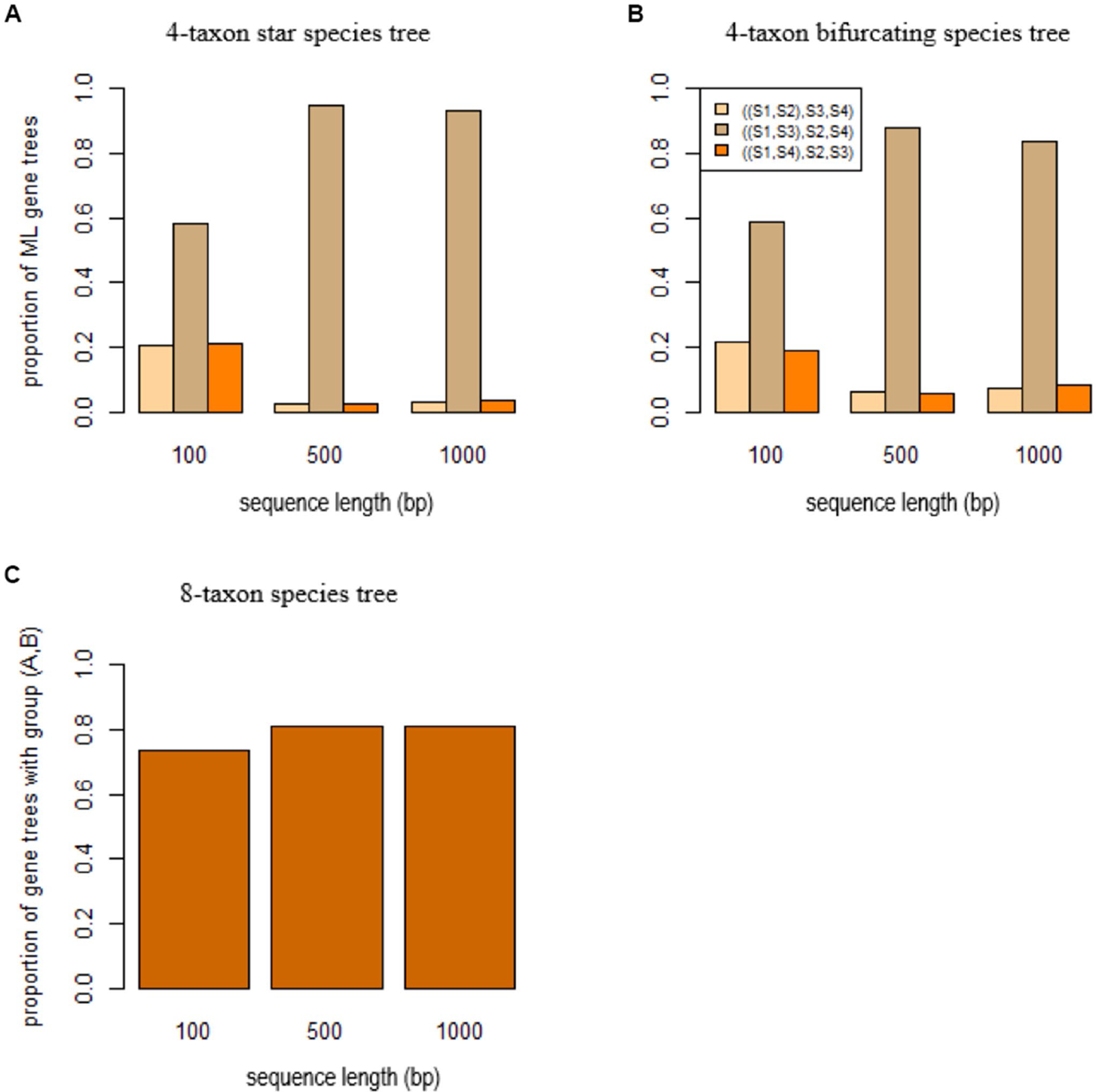
Short branch attraction in species trees. ML gene trees were built by RAxML for the sequences simulated from **(A)** the 4-taxon star species tree ($S_{1}:0.0001$, $S_{2}:0.01$, $S_{3}:0.0001$, $S_{4}:0.01$), **(B)** the 4-taxon bifurcating species tree (($\left(S_{1}:0.0001,S_{2}:0.01\right):0.0001$, $S_{3}:0.0001$): 0.0001, $S_{4}:0.01$), and **(C)** the 8-taxon species tree ((A:0.0001, ($\left({\text{S}}_{1}:0.01,{\text{S}}_{2}:0.01\right):0.01$, ${\text{S}}_{3}:0.02$): 0.01): 0.0001, B: 0.0001, ($\left({\text{S}}_{4}:0.01,{\text{S}}_{5}:0.01\right):0.01$, ${\text{S}}_{6}:0.02$): 0.01) with two short branches leading to the species A and B. The x-axis is the sequence length 100, 500, and 1,000bp.

To investigate the effect of SBA on species tree estimation, gene trees were simulated from three species trees: a 4-taxon star species tree ($S_{1}:0.0001$, $S_{2}:0.01$, $S_{3}:0.0001$, $S_{4}:0.01$), a 4-taxon bifurcating species tree(($\left(S_{1}:0.0001,S_{2}:0.01\right):0.0001$, $S_{3}:0.0001$):0.0001, $S_{4}:0.01$), and an 8-taxon species tree (((A:0.0001, ($\left({\text{S}}_{1}:0.01,{\text{S}}_{2}:0.01\right):0.01$, ${\text{S}}_{3}:0.02$): 0.01): 0.0001, B: 0.0001):0.0001, ($\left({\text{S}}_{4}:0.01,{\text{S}}_{5}:0.01\right):0.01$, ${\text{S}}_{6}:0.02$): 0.01). The 4 -taxon species trees include two short branches leading to species $S_{1}$ and $S_{2}$, while species A and B have a short branch in the 8-taxon species tree. Under the coalescent model, three (unrooted) gene trees $\left(\left(S_{1},S_{2}\right),S_{3},S_{4}\right)$, $\left(\left(S_{1},S_{3}\right),S_{2},S_{4}\right)$, and $\left(\left(S_{1},S_{4}\right),S_{2},S_{3}\right)$ can be generated from the 4-taxon species tree and they are expected to have equal probabilities $\left(\frac{1}{3},\frac{1}{3},\frac{1}{3}\right)$ when the species tree is a star tree. Accordingly, if the true gene trees are given, coalescent methods are expected to estimate three (unrooted) species trees with equal probabilities $\left(\frac{1}{3},\frac{1}{3},\frac{1}{3}\right)$. However, due to the SBA, the ML gene trees estimated from DNA sequences tend to group the species ${\text{S}}_{1}$ and ${\text{S}}_{3}$ across genes, resulting in a high proportion (58%, 94, 93% for the sequence length 100 bp, 500 bp, and 1,000 bp, respectively, Figure 4A) of the tree $\left(\left(S_{1},S_{3}\right),S_{2},S_{4}\right)$. As a result, the coalescent methods estimated the wrong species tree $\left(\left(S_{1},S_{3}\right),S_{2},S_{4}\right)$. For the 4-taxon bifurcating species tree, a high proportion of the ML gene trees support the tree $\left(\left(S_{1},S_{3}\right),S_{2},S_{4}\right)$ (Figure 4B). Again, the coalescent methods, misled by the biased ML gene trees, estimated the wrong species tree $\left(\left(S_{1},S_{3}\right),S_{2},S_{4}\right)$. Similarly, SBA significantly biased the gene tree distribution (i.e., >70% of the ML gene trees comprise a group (A, B)) (Figure 4C) for the 8-taxon species tree, and the coalescent methods estimated the wrong species tree containing a monophyletic group (A, B) of the species A and B.

## 3. Materials and methods

### 3.1. The algorithmic bias of PhyML and RAxML

#### 3.1.1. Scenario 1: identical sequences

A sequence of 100, 500, 1,000 nucleotides were randomly generated from the multinomial distribution with probabilities $p_{A}=p_{C}=p_{G}=p_{T}=0.25$. Then, the sequence was replicated four times to generate identical sequences for species $S_{1}$, $S_{2}$, $S_{3}$, and $S_{4}$. Phylogenetic trees were estimated from four identical sequences using the phylogenetic programs PhyML and RAxML with the GTRGAMMA substitution model (Yang, 2006). Each simulation was repeated 100 times. The proportions of three bifurcating trees $tree1=\left(\left(S_{1},S_{2}\right),S_{3},S_{4}\right)$, $tree2=\left(\left(S_{1},S_{3}\right),S_{2},S_{4}\right)$, and $tree3=\left(\left(S_{1},S_{4}\right),S_{2},S_{3}\right)$ were calculated and compared with the expected probabilities $\left(1/3,1/3,1/3\right)$ using the multinomial test.

#### 3.1.2. Scenario 2: saturated sequences

A sequence of 100, 500, 1,000 nucleotides were generated independently from the multinomial distribution with probabilities $P_{A}=0.1$, $P_{C}=0.2$, $P_{G}=0.3$, $P_{T}=0.4$ for species $S_{1}$, $S_{2}$, $S_{3}$, and $S_{4}$. Phylogenetic trees were estimated from the four saturated sequences using PhyML and RAxML with the GTRGAMMA substitution model. Each simulation was repeated 100 times. The proportions of three bifurcating trees $tree1=\left(\left(S_{1},S_{2}\right),S_{3},S_{4}\right)$, $tree2=\left(\left(S_{1},S_{3}\right),S_{2},S_{4}\right)$, and $tree3=\left(\left(S_{1},S_{4}\right),S_{2},S_{3}\right)$ were calculated and compared with the expected probabilities $\left(1/3,1/3,1/3\right)$ using the multinomial hypothesis test. To investigate the effects of missing characters on the algorithmic bias of PhyML and RAxML, 10% of the nucleotides were removed at random from the saturated sequences for species $S_{1}$ and $S_{2}$. Then, phylogenetic trees were reconstructed and the proportions of three bifurcating trees were calculated and compared with the expected probabilities $\left(1/3,1/3,1/3\right)$ using the multinomial hypothesis test.

#### 3.1.3. Scenario 3: sequences generated from a 4-taxon star tree

DNA sequences of 100, 500, 1,000 bps were generated from a 4-taxon star tree ($S_{1}:0.01$, $S_{2}:0.01$, $S_{3}:0.01$, $S_{4}:0.01$. The sequences were simulated using a phylogenetic program Seq-Gen (Rambaut and Grassly, 1997) with the substitution model GTR + GAMMA where the base frequencies were simulated from the Dirichlet distribution $\left(1,1,1,1\right)$, and the rate parameters were simulated from the lognormal distribution $\left(6,1,1\right)$, and the shape parameter was simulated from the normal distribution with mean 0.5 and variance 0.01. Phylogenetic trees were estimated by PhyML and RAxML with the GTRGAMMA substitution model. The PhyML command line for building ML phylogenetic trees is *phyml -i input -ae -b0 -mGTR*. The RAxML command line for building ML trees is *raxml-HPC-SSE3 -s input -n output -mGTRGAMMA -p random_seed*.

### 3.2. Short branch attraction in gene trees

#### 3.2.1. Scenario 1: 4-taxon star trees with unequal branch lengths

DNA sequences of 100, 500, and 1,000 bps were generated from two 4 -taxon star trees. The first star tree ($S_{1}:0.0001$, $S_{2}:0.01$, $S_{3}:0.0001$, $S_{4}:0.01$) has two short branches of length 0.0001 leading to the species $S_{1}$ and $S_{3}$. The second star tree is tree ($S_{1}:0.1$, $S_{2}:0.01$, $S_{3}:0.1$, $S_{4}:0.01$) in which the length of two short branches in the first tree increases from 0.0001 to 0.1. To generate nucleotides under the GTR + GAMMA substitution model, the base frequencies were simulated from the Dirichlet distribution $\left(1,1,1,1\right)$; the rate parameters were simulated from the lognormal distribution $\left(6,1,1\right)$; the shape parameter was simulated from the normal distribution with mean 0.5 and variance 0.01. The ML phylogenetic trees were estimated by RAxML with the GTRGAMMA substitution model using the same command line in the previous section.

#### 3.2.2. Scenario 2: 4-taxon bifurcating trees with unequal branch lengths

DNA sequences were generated from two 4-taxon bifurcating trees with unequal branch lengths. The first 4-taxon tree ($\left(S_{1}:0.0001,S_{2}:0.01\right):0.0001$, $S_{3}:0.0001$, $S_{4}:0.01$) has a short internal branch and two short terminal branches leading to species $S_{1}$ and $S_{3}$. The second 4-taxon bifurcating tree is ($\left(S_{1}:0.1,S_{2}:0.01\right):0.0001$, $S_{3}:0.1$, $S_{4}:0.01$) in which the length of two short branches in the first 4-taxon tree increases from 0.0001 to 0.1 DNA sequences of 100,500, and 1,000 bps were simulated under the substitution model GTR + GAMMA where the base frequencies were simulated from the Dirichlet distribution $\left(1,1,1,1\right)$, and the rate parameters were simulated from the lognormal distribution $\left(6,1,1\right)$, and the shape parameter was simulated from the normal distribution with mean 0.5 and variance 0.01. Phylogenetic trees were estimated by PhyML and RAxML with the substitution model GTRGAMMA substitution model.

#### 3.2.3. Scenario 3: n-taxon trees

DNA sequences of 100, 500, and 1,000 bps were generated from an 8-taxon tree ((A: 0.0001, ($\left({\text{S}}_{1}:0.01,{\text{S}}_{2}:0.01\right):0.01$, ${\text{S}}_{3}:0.01$): 0.01): 0.0001, B: 0.0001, ($\left({\text{S}}_{4}:0.01,{\text{S}}_{5}:0.01\right):0.01$, ${\text{S}}_{6}:0.01$): 0.01). The ML trees were built by RAxML with the GTRGAMMA model. The simulation was repeated 100 times and we calculated the proportion of the ML trees containing the monophyletic group of the species A and B (the evidence for SBA in the 8-taxon tree).

### 3.3. Short branch attraction in species trees

DNA sequences were simulated from three non-clock species trees. The first species tree is a 4-taxon star tree($S_{1}:0.0001$, $S_{2}:0.01$, $S_{3}:0.0001$, $S_{4}:0.01$) with two short branches $\left(\text{length}=0.0001\right)$ leading to the species S1 and S3. The population size parameter in the root population is set to θ=0.0001. The second species tree is a 4-taxon bifurcating tree (($\left(S_{1}:0.0001,S_{2}:0.01\right):0.0001$, $S_{3}:0.0001$): 0.0001, $S_{4}:0.01$) with two short internal branches $\left(\text{length}=0.0001\right)$ and two short terminal branches $\left(\text{length}=0.0001\right)$ leading to the species $S_{1}$ and $S_{3}$. The population size parameters θ=0.0001 for the root population and θ=0.01 for the other two internal branches (i.e., ancestral populations). The third species tree is an 8-taxon tree (((A: 0.0001, ($\left({\text{S}}_{1}:0.01,{\text{S}}_{2}:0.01\right):0.01$, ${\text{S}}_{3}:0.02$): 0.01): 0.0001, B: 0.0001):0.0001, ($\left({\text{S}}_{4}:0.01,{\text{S}}_{5}:0.01\right):0.01$, ${\text{S}}_{6}:0.02$): 0.01) with two short branches leading to the species A and B. The population size parameters θ=0.0001 for the root population and θ=0.01 for the other internal branches (i.e., ancestral populations) in the 8-taxon species tree. One thousand gene trees were generated from each of the three species trees under the multispecies coalescent model using an R package Phybase (Liu and Yu, 2010). Then, DNA sequences were simulated from the gene trees using the GTR + GAMMA substitution model where the base frequencies were simulated from the Dirichlet distribution $\left(1,1,1,1\right)$, and the rate parameters were simulated from the lognormal distribution $\left(6,1,1\right)$, and the shape parameter was simulated from the normal distribution with mean 0.5 and variance 0.01. The ML gene trees were built from the simulated DNA sequences by RAxML and then were compared with the true gene trees simulated from the three species trees. Finally, the species trees were estimated from the ML gene trees using a coalescent method NJst (Liu and Yu, 2011).

## 4. Discussion

Under regularity conditions (absolute continuity, identifiability, etc.), the MLEs are statistically consistent in estimating model parameters as the sample size goes to infinity. The estimator $\hat{\theta }$ of the parameter θ is said to be a biased estimator if the expected value of $\hat{\theta }$ is not equal to the true parameter value θ, i.e., $E\left(\hat{\theta }\right)\neq \theta$. When the sample size is finite, it is frequently observed that the MLE ${\hat{\theta }}_{MLE}$ is a biased estimator of the model parameter θ. Hence, it is important to investigate/explore the behavior of MLEs in the context of a finite sample size. The theory developed in this paper indicates that the presence of heterogeneous branch lengths (i.e., SBA) can introduce bias into the probability distribution of the ML gene trees, consequently leading to erroneous species tree inference. The findings align with a prior investigation (Dornburg et al., 2019) which demonstrated that the utility of a character depends on the relative rates and times of evolution of subtending lengths of the internode to be resolved and therefore heterogeneous branch lengths can introduce bias into phylogenetic inference. SBA may occur in the polytomy of trees and in bifurcating trees with two short terminal branches separated by a similarly short internal branch. Our theoretical and simulation analyses surprisingly demonstrate that short internal branches not only introduce a large amount of phylogenetic uncertainty but can also severely bias gene tree and species tree inference. The simulation for identical sequences, saturated sequences, and phylogenetic trees with polytomies suggests that widely applied phylogenetic programs PhyML and RAxML favor a particular bifurcating tree, rather than producing three equally likely bifurcating trees. PhyML appears to be more problematic than RAxML in estimating phylogenetic trees from noninformative sequences. The algorithmic artefact of PhyML and RAxML may further exacerbate the effect of SBA on gene tree and species tree estimation.

LBA is a phenomenon in molecular phylogenetics where rapidly evolving lineages tend to cluster together in a phylogenetic tree, even if they are not actually closely related. It can lead to erroneous inferences of evolutionary relationships if distantly related lineages share many rapidly evolving characters due to substitution saturation. Analytic tools have been developed to detect and avoid the LBA artifact (Su and Townsend, 2015). One way to address the problems of LBA is to use more slowly evolving characters or to use more complex models that can account for the different rates of evolution among different lineages. Another approach is to shorten long branches by increasing taxon sampling. In contrast, SBA artifacts can create an “artificial” branch length that connects distantly related taxa when their molecular sequences share numerous slowly evolving characters due to short branches. Presently, there are no effective techniques to counteract the adverse impacts of the SBA artifact on gene and species tree estimation.

We hypothesize that trees with short internal branches may lead to SBA, because the sequence data generated from such trees lack sufficient phylogenetic information to resolve the corresponding short internal branches. Likewise, there is a lack of phylogenetic information in the identical and saturated sequences, or the sequences generated from star trees, causing the algorithmic error of PhyML and RAxML. Thus, lack of phylogenetic signals is the primary cause of SBA and the algorithmic bias of phylogenetic programs PhyML and RAxML. We previously demonstrated similar pathological results involving gene tree estimation when short gene sequences with minimal phylogenetic data were involved (Xi et al., 2016). In particular, relationships were resolved artificially by taxon ordering in the data matrix leading to spurious species tree inference. Since SBA is caused by a lack of phylogenetic information, removing short loci from phylogenetic analysis can lower the bias in gene trees and species tree estimation, but a decline in the number of loci can increase the uncertainty of species tree estimation.

The multispecies coalescent model is a hierarchical model involving two stochastic processes: mutation process and coalescence. The mutation process describes how nucleotides evolve in gene trees, whereas the coalescence process describes how genealogical lineages evolve in the species tree. The algorithmic bias and systematic error due to SBA we uncovered here are rooted in the mutation process and only affect gene tree estimation. Because accurate estimation of species trees relies on accurate estimation of gene trees, biased gene trees can mislead species tree estimation. Moreover, LBA and SBA artifacts are the consequence of minimal phylogenetic signal in the sequence data, rather than deficiencies involving multispecies coalescent models. Our simulation indicates that 95% of the biased gene trees have an extremely short internal branch $\left(\text{length}<1\text{e}-5\right)$. This suggests that converting these short branches to a polytomy in the estimated gene trees can likely reduce the algorithmic bias and SBA artifacts. We have developed an algorithm in the latest version of MP-EST (Liu et al., 2010) to convert short branches to a polytomy in the ML gene trees with a plan to update the species tree estimation program MP-EST such that it can take polytomy gene trees to reconstruct species trees.

## Supplementary Material

Short Branch Supp

## Data Availability

The datasets presented in this study can be found in online repositories. The names of the repository/repositories and accession number(s) can be found at: https://doi.org/10.6084/m9.figshare.21793109.
