# Supplementary material for: Short branch attraction in phylogenomic inference under the multispecies coalescent
Source: Front Ecol Evol. Author manuscript; Available in PMC 2024 Sep 4. (PMC11372852; doi:10.3389/fevo.2023.1134764)
Supplement: Short Branch Supp [file NIHMS2011024-supplement-Short_Branch_Supp.pdf]

Short Branch Attraction in Phylogenomic Inference Under the Multispecies Coalescent

Appendix

Consider a bifurcating 4-taxon tree with branch lengths t_0, t_1, t_2, t_3, t_4 where t_0 is the length of the internal branch and $t_i's$ for $i > 0$ are the lengths of four terminal branches. Let x_{n_1} and x_{n_2} be the nucleotides at two internal nodes. Under the Jukes-Cantor model, the probability $P_{XY}(t) = \begin{cases} \frac{1}{4}+\frac{3}{4}e^{-\frac{4t}{3}} \; if \; X = Y \\ \frac{1}{4}-\frac{1}{4}e^{-\frac{4t}{3}} \; if \; X \neq Y \end{cases}$. To simply the notation, we use p_1^i to denote the probability $P_{XY}(t_i)$ for $X = Y$ and we use p_2^i to denote the probability $P_{XY}(t_i)$ for $X \neq Y$. Here, we assume $0 \leq t_1 \leq t_2 \leq t_3 \leq t_4 < \infty$. Furthermore, it can be shown that

1. $p_1^i > p_2^i$ for $i = 1, 2, 3, 4$
2. If $i < j$, then $p_1^i > p_1^j$
3. If $i < j$, then $p_2^i < p_2^j$

A1: Consider a 4-taxon star tree with branch length $\boldsymbol{0 < t_1 \leq t_2 \leq t_3 \leq t_4 < \infty}$. The probability of the site pattern $\boldsymbol{xxxy}$ is greater than the probability of the site pattern $\boldsymbol{xxyy}$, i.e., $\boldsymbol{P(xxxy) + P(xxyx) + P(xyxx) + P(yxxx) > P(xxyy) + P(xyyx) + P(xyxy)}$.

Proof: There are 12 combinations of 4 nucleotides that fit the site patterns $xxxy$, $xxyx$, $xyxx$, $yxxx$, $xxyy$, $xyyx$, $xyxy$ respectively. Also, the 12 combinations for the site pattern $xxxy$ (or the other site patterns) have the same probability. Thus, it is sufficient to show that $P(AAAC) + P(AAAC) + P(AACA) + P(ACAA) + P(CAAA) > \big(P(AACC) + P(ACAC) + P(ACCA)\big)$. We first find the probability of the site patterns with three identical characters,

$P(AAAC)+P(AACA)+P(ACAA)+P(CAAA)$

$$=\frac{1}{4}\big(P_{AA}(t_1)P_{AA}(t_2)P_{AA}(t_3)P_{AC}(t_4)+P_{CA}(t_1)P_{CA}(t_2)P_{CA}(t_3)P_{CC}(t_4)$$

$$+P_{GA}(t_1)P_{GA}(t_2)P_{GA}(t_3)P_{GC}(t_4)+P_{TA}(t_1)P_{TA}(t_2)P_{TA}(t_3)P_{TC}(t_4)$$

$$+P_{AA}(t_1)P_{AA}(t_2)P_{AC}(t_3)P_{AA}(t_4)+P_{CA}(t_1)P_{CA}(t_2)P_{CC}(t_3)P_{CA}(t_4)$$

$$+P_{GA}(t_1)P_{GA}(t_2)P_{GC}(t_3)P_{GA}(t_4)+P_{TA}(t_1)P_{TA}(t_2)P_{TC}(t_3)P_{TA}(t_4)\big)$$

$$=\frac{1}{4}(p_1^1p_1^2p_1^3p_2^4+p_1^1p_1^2p_2^3p_1^4+p_1^1p_2^2p_1^3p_1^4+p_2^1p_1^2p_1^3p_1^4+p_1^1p_2^2p_2^3p_2^4+p_2^1p_1^2p_2^3p_2^4$$

$$+p_2^1p_2^2p_1^3p_2^4+p_2^1p_2^2p_2^3p_1^4+8p_2^1p_2^2p_2^3p_2^4\big)\ (1)$$

and

$P(AACC)+P(ACAC)+P(ACCA)$

$$=\frac{1}{4}\big(P_{AA}(t_1)P_{AA}(t_2)P_{AC}(t_3)P_{AC}(t_4)+P_{CA}(t_1)P_{CA}(t_2)P_{CC}(t_3)P_{CC}(t_4)$$

$$+P_{GA}(t_1)P_{GA}(t_2)P_{GC}(t_3)P_{GC}(t_4)+P_{TA}(t_1)P_{TA}(t_2)P_{TC}(t_3)P_{TC}(t_4)\big)$$

$$=\frac{1}{4}(p_1^1p_1^2p_2^3p_2^4+p_1^1p_2^2p_1^3p_2^4+p_1^1p_2^2p_2^3p_1^4+p_2^1p_1^2p_1^3p_2^4+p_2^1p_1^2p_2^3p_1^4+p_2^1p_2^2p_1^3p_1^4$$

$$+6p_2^1p_2^2p_2^3p_2^4)\ (2)$$

Note that we have

$$p_1^1p_1^2p_1^3p_2^4+p_1^1p_2^2p_2^3p_2^4-(p_1^1p_1^2p_2^3p_2^4+p_1^1p_2^2p_1^3p_2^4)=p_1^1p_2^4(p_1^2-p_2^2)(p_1^3-p_2^3)>0$$

$$p_1^1p_1^2p_2^3p_1^4+p_2^1p_2^2p_2^3p_1^4-(p_2^1p_1^2p_2^3p_1^4+p_1^1p_2^2p_2^3p_1^4)=p_2^3p_1^4(p_1^1-p_2^1)(p_1^2-p_2^2)>0$$

$$p_2^1p_1^2p_1^3p_1^4+p_2^1p_2^2p_1^3p_2^4-(p_2^1p_1^2p_1^3p_2^4+p_2^1p_2^2p_1^3p_1^4)=p_2^1p_1^3(p_1^2-p_2^2)(p_1^4-p_2^4)>0$$

Thus,

$P(AAAC) + P(AACA) + P(ACAA) + P(CAAA) - \big(P(AACC) + P(ACAC) + P(ACCA)\big)$

$$= \frac{1}{4}(p_1^1 p_1^2 p_1^3 p_2^4 + p_1^1 p_1^2 p_2^3 p_1^4 + p_1^1 p_2^2 p_1^3 p_1^4 + p_2^1 p_1^2 p_1^3 p_1^4 + p_1^1 p_2^2 p_2^3 p_2^4 + p_2^1 p_1^2 p_2^3 p_2^4$$

$$+ p_2^1 p_2^2 p_1^3 p_2^4 + p_2^1 p_2^2 p_2^3 p_1^4 + 8 p_2^1 p_2^2 p_2^3 p_2^4)$$

$$- \frac{1}{4}(p_1^1 p_1^2 p_2^3 p_2^4 + p_1^1 p_2^2 p_1^3 p_2^4 + p_1^1 p_2^2 p_2^3 p_1^4 + p_2^1 p_1^2 p_1^3 p_2^4 + p_2^1 p_1^2 p_2^3 p_1^4 + p_2^1 p_2^2 p_1^3 p_1^4$$

$$+ 6 p_2^1 p_2^2 p_2^3 p_2^4) > 0$$

■

A2: Consider a 4-taxon star tree with branch length $t_1, t_2 < t_3, t_4$. Then, $P(xxyy) > P(xyyx)$ and $P(xxyy) > P(xyxy)$.

Proof: There are 12 combinations of 4 nucleotides that fit the site patterns $xxyy, xyyx, xyxy$. The 12 combinations have the same probability under the Jukes-Cantor substitution model. Hence, it suffices to show that $P(AACC) > P(ACAC)$ and $P(AACC) > P(ACCA)$.

$$P(AACC) = P_{AA}(t_1)P_{AA}(t_2)P_{AC}(t_3)P_{AC}(t_4) + P_{CA}(t_1)P_{CA}(t_2)P_{CC}(t_3)P_{CC}(t_4)$$
$$+ P_{GA}(t_1)P_{GA}(t_2)P_{GC}(t_3)P_{GC}(t_4) + P_{TA}(t_1)P_{TA}(t_2)P_{TC}(t_3)P_{TC}(t_4)$$
$$= P_1^1 P_1^2 P_2^3 P_2^4 + P_2^1 P_2^2 P_1^3 P_1^4 + 2 P_2^1 P_2^2 P_2^3 P_2^4$$

$$P(ACAC) = P_{AA}(t_1)P_{AC}(t_2)P_{AA}(t_3)P_{AC}(t_4) + P_{CA}(t_1)P_{CC}(t_2)P_{CA}(t_3)P_{CC}(t_4)$$
$$+ P_{GA}(t_1)P_{GC}(t_2)P_{GA}(t_3)P_{GC}(t_4) + P_{TA}(t_1)P_{TC}(t_2)P_{TA}(t_3)P_{TC}(t_4)$$
$$= P_1^1 P_2^2 P_1^3 P_2^4 + P_2^1 P_1^2 P_2^3 P_1^4 + 2 P_2^1 P_2^2 P_2^3 P_2^4$$

$$P(ACCA) = P_{AA}(t_1)P_{AC}(t_2)P_{AC}(t_3)P_{AA}(t_4) + P_{CA}(t_1)P_{CC}(t_2)P_{CC}(t_3)P_{CA}(t_4)$$
$$+ P_{GA}(t_1)P_{GC}(t_2)P_{GC}(t_3)P_{GA}(t_4) + P_{TA}(t_1)P_{TC}(t_2)P_{TC}(t_3)P_{TA}(t_4)$$
$$= P_1^1 P_2^2 P_2^3 P_1^4 + P_2^1 P_1^2 P_1^3 P_2^4 + 2 P_2^1 P_2^2 P_2^3 P_2^4$$

Thus, $P(AACC) - P(ACAC) = P_1^1 P_1^2 P_2^3 P_2^4 + P_2^1 P_2^2 P_1^3 P_1^4 + 2 P_2^1 P_2^2 P_2^3 P_2^4 - P_1^1 P_2^2 P_1^3 P_2^4 - P_2^1 P_1^2 P_2^3 P_1^4 - 2 P_2^1 P_2^2 P_2^3 P_2^4 > P_2^1 P_1^2 P_2^3 P_1^4 + P_2^1 P_2^2 P_1^3 P_1^4 - P_2^1 P_2^2 P_1^3 P_1^4 - P_2^1 P_1^2 P_2^3 P_1^4 = 0$, and

$P(AACC) - P(ACCA) = P_1^1 P_1^2 P_2^3 P_2^4 + P_2^1 P_2^2 P_1^3 P_1^4 + 2P_2^1 P_2^2 P_2^3 P_2^4 - P_1^1 P_2^2 P_2^3 P_1^4 - P_2^1 P_1^2 P_1^3 P_2^4 - 2P_2^1 P_2^2 P_2^3 P_2^4 > P_2^1 P_1^2 P_1^3 P_2^4 + P_2^1 P_2^2 P_1^3 P_1^4 - P_2^1 P_2^2 P_1^3 P_1^4 - P_2^1 P_1^2 P_1^3 P_2^4 = 0$

■

A3: If the nucleotides at two internal nodes of a bifurcating 4-taxon tree are distinct, the probability of the site pattern $xxyy$ is greater than the probability of the site pattern $xyxy$, i.e., $P(xxyy) > P(xyxy)$. Similarly, $P(xxyy) > P(xyyx)$.

Proof: There are 12 combinations of 4 nucleotides that fit the site pattern $xxyy$. Similarly, there are 12 combinations for the site pattern $xyxy$. The 12 combinations have the same probability under the Jukes-Cantor substitution model. Hence, it suffices to show that the probability of $AACC$ is greater than the probability of $ACAC$. If two nucleotides at the internal nodes are distinct, the probability of AACC is given by

$$\begin{aligned}
P(AACC) = {} & P_{AA}(t_1)P_{AA}(t_2)P_{CC}(t_3)P_{CC}(t_4) + P_{AA}(t_1)P_{AA}(t_2)P_{GC}(t_3)P_{GC}(t_4) \\
& + P_{AA}(t_1)P_{AA}(t_2)P_{TC}(t_3)P_{TC}(t_4) + P_{CA}(t_1)P_{CA}(t_2)P_{AC}(t_3)P_{AC}(t_4) \\
& + P_{CA}(t_1)P_{CA}(t_2)P_{GC}(t_3)P_{GC}(t_4) + P_{CA}(t_1)P_{CA}(t_2)P_{TC}(t_3)P_{TC}(t_4) \\
& + P_{GA}(t_1)P_{GA}(t_2)P_{AC}(t_3)P_{AC}(t_4) + P_{GA}(t_1)P_{GA}(t_2)P_{CC}(t_3)P_{CC}(t_4) \\
& + P_{GA}(t_1)P_{GA}(t_2)P_{TC}(t_3)P_{TC}(t_4) + P_{TA}(t_1)P_{TA}(t_2)P_{AC}(t_3)P_{AC}(t_4) \\
& + P_{TA}(t_1)P_{TA}(t_2)P_{CC}(t_3)P_{CC}(t_4) + P_{TA}(t_1)P_{TA}(t_2)P_{GC}(t_3)P_{GC}(t_4) \\
& = P_1^1 P_1^2 P_1^3 P_1^4 + 2P_1^1 P_1^2 P_2^3 P_2^4 + 7P_2^1 P_2^2 P_2^3 P_2^4 + 2P_2^1 P_2^2 P_1^3 P_1^4
\end{aligned}$$

The probability of $ACAC$ is given by

$$
\begin{aligned}
P(ACAC) = {} & P_{AA}(t_1)P_{AC}(t_2)P_{CA}(t_3)P_{CC}(t_4) + P_{AA}(t_1)P_{AC}(t_2)P_{GA}(t_3)P_{GC}(t_4) \\
& + P_{AA}(t_1)P_{AC}(t_2)P_{TA}(t_3)P_{TC}(t_4) + P_{CA}(t_1)P_{CC}(t_2)P_{AA}(t_3)P_{AC}(t_4) \\
& + P_{CA}(t_1)P_{CC}(t_2)P_{GA}(t_3)P_{GC}(t_4) + P_{CA}(t_1)P_{CC}(t_2)P_{TA}(t_3)P_{TC}(t_4) \\
& + P_{GA}(t_1)P_{GC}(t_2)P_{AA}(t_3)P_{AC}(t_4) + P_{GA}(t_1)P_{GC}(t_2)P_{CA}(t_3)P_{CC}(t_4) \\
& + P_{GA}(t_1)P_{GC}(t_2)P_{TA}(t_3)P_{TC}(t_4) + P_{TA}(t_1)P_{TC}(t_2)P_{AA}(t_3)P_{AC}(t_4) \\
& + P_{TA}(t_1)P_{TC}(t_2)P_{CA}(t_3)P_{CC}(t_4) + P_{TA}(t_1)P_{TC}(t_2)P_{GA}(t_3)P_{GC}(t_4) \\
& = P_1^1P_2^2P_2^3P_1^4 + 2P_1^1P_2^2P_2^3P_2^4 + 2P_2^1P_2^2P_2^3P_2^4 + 3P_2^1P_2^2P_1^3P_2^4 + 2P_2^1P_1^2P_2^3P_2^4 \\
& + 2P_2^1P_2^2P_2^3P_1^4
\end{aligned}
$$

The probability of $ACCA$ is given by

$$
\begin{aligned}
P(ACCA) = {} & P_{AA}(t_1)P_{AC}(t_2)P_{CC}(t_3)P_{CA}(t_4) + P_{AA}(t_1)P_{AC}(t_2)P_{GC}(t_3)P_{GA}(t_4) \\
& + P_{AA}(t_1)P_{AC}(t_2)P_{TC}(t_3)P_{TA}(t_4) + P_{CA}(t_1)P_{CC}(t_2)P_{AC}(t_3)P_{AA}(t_4) \\
& + P_{CA}(t_1)P_{CC}(t_2)P_{GC}(t_3)P_{GA}(t_4) + P_{CA}(t_1)P_{CC}(t_2)P_{TC}(t_3)P_{TA}(t_4) \\
& + P_{GA}(t_1)P_{GC}(t_2)P_{AC}(t_3)P_{AA}(t_4) + P_{GA}(t_1)P_{GC}(t_2)P_{CC}(t_3)P_{CA}(t_4) \\
& + P_{GA}(t_1)P_{GC}(t_2)P_{TC}(t_3)P_{TA}(t_4) + P_{TA}(t_1)P_{TC}(t_2)P_{AC}(t_3)P_{AA}(t_4) \\
& + P_{TA}(t_1)P_{TC}(t_2)P_{CC}(t_3)P_{CA}(t_4) + P_{TA}(t_1)P_{TC}(t_2)P_{GC}(t_3)P_{GA}(t_4) \\
& = P_1^1P_2^2P_1^3P_2^4 + P_2^1P_1^2P_2^3P_1^4 + 2P_1^1P_2^2P_2^3P_2^4 + 2P_2^1P_2^2P_2^3P_2^4 + 2P_2^1P_2^2P_1^3P_2^4 \\
& + 2P_2^1P_1^2P_2^3P_2^4 + 2P_2^1P_2^2P_2^3P_1^4
\end{aligned}
$$

We first show that $P(AACC) > P(ACAC)$. Observe that

$$P_2^1P_2^2P_1^3P_1^4 + P_2^1P_2^2P_2^3P_2^4 - P_2^1P_2^2P_2^3P_1^4 - P_2^1P_2^2P_1^3P_2^4 = P_2^1P_2^2(P_1^3 - P_2^3)(P_1^4 - P_2^4) > 0$$

and

$$P_1^1P_1^2P_2^3P_2^4 + P_2^1P_2^2P_2^3P_2^4 - P_1^1P_2^2P_2^3P_2^4 - P_2^1P_1^2P_2^3P_2^4 = P_2^3P_2^4(P_1^1 - P_2^1)(P_1^2 - P_2^2) > 0$$

and

$$P_1^1P_1^2P_1^3P_1^4 + P_2^1P_2^2P_2^3P_2^4 - P_1^1P_2^2P_2^3P_1^4 - P_2^1P_2^2P_1^3P_2^4$$
$$> P_2^1P_1^2P_1^3P_2^4 + P_2^1P_2^2P_2^3P_2^4 - P_2^1P_2^2P_2^3P_2^4 - P_2^1P_2^2P_1^3P_2^4 > 0$$

and

$$P_1^1P_1^2P_1^3P_1^4 + P_2^1P_2^2P_2^3P_2^4 - P_1^1P_2^2P_1^3P_2^4 - P_2^1P_1^2P_2^3P_1^4 = (P_1^1P_1^3 - P_2^1P_2^3)(P_1^2P_1^4 - P_2^2P_2^4) > 0$$

Thus,

$$P(AACC) = P_1^1P_1^2P_1^3P_1^4 + 2P_1^1P_1^2P_2^3P_2^4 + 7P_2^1P_2^2P_2^3P_2^4 + 2P_2^1P_2^2P_1^3P_1^4$$
$$> P_1^1P_2^2P_2^3P_1^4 + 2P_1^1P_2^2P_2^3P_2^4 + 2P_2^1P_2^2P_2^3P_2^4 + 3P_2^1P_2^2P_1^3P_2^4 + 2P_2^1P_1^2P_2^3P_2^4$$
$$+ 2P_2^1P_2^2P_2^3P_1^4 = P(ACAC)$$

and

$$P(AACC) = P_1^1P_1^2P_1^3P_1^4 + 2P_1^1P_1^2P_2^3P_2^4 + 7P_2^1P_2^2P_2^3P_2^4 + 2P_2^1P_2^2P_1^3P_1^4$$
$$> P_1^1P_2^2P_1^3P_2^4 + P_2^1P_1^2P_2^3P_1^4 + 2P_1^1P_2^2P_2^3P_2^4 + 2P_2^1P_2^2P_2^3P_2^4 + 2P_2^1P_2^2P_1^3P_2^4$$
$$+ 2P_2^1P_1^2P_2^3P_2^4 + 2P_2^1P_2^2P_2^3P_1^4 = P(ACCA)$$

We have shown that $P(AACC) > P(ACAC)$ and $(AACC) > P(ACCA)$. It follows that $P(xxyy) = 12P(AACC) > 12P(ACAC) = P(xyxy)$ and $P(xxyy) = 12P(AACC) > 12P(ACCA) = P(xyyx)$. Note that the inequalities $P(xxyy) > P(xyxy)$ and $P(xxyy) > P(xyyx)$ do not depend on the order of branch lengths t_1, t_2, t_3, t_4. Thus, $P(xxyy) > P(xyxy)$ and $P(xxyy) > P(xyyx)$ for all bifurcating 4-taxon trees.

■
